# Bone Regeneration Induced by Patient-Adapted Mg Alloy-Based Scaffolds for Bone Defects: Present and Future Perspectives

**DOI:** 10.3390/biomimetics8080618

**Published:** 2023-12-17

**Authors:** Veronica Manescu (Paltanea), Iulian Antoniac, Aurora Antoniac, Dan Laptoiu, Gheorghe Paltanea, Robert Ciocoiu, Iosif Vasile Nemoianu, Lucian Gheorghe Gruionu, Horatiu Dura

**Affiliations:** 1 Faculty of Material Science and Engineering, National University of Science and Technology Politehnica Bucharest, 313 Splaiul Independentei, District 6, RO-060042 Bucharest, Romania; veronica.paltanea@upb.ro (V.M.); antoniac.aurora@gmail.com (A.A.); ciocoiurobert@gmail.com (R.C.); 2 Faculty of Electrical Engineering, National University of Science and Technology Politehnica Bucharest, 313 Splaiul Independentei, District 6, RO-060042 Bucharest, Romania; gheorghe.paltanea@upb.ro (G.P.); iosif.nemoianu@upb.ro (I.V.N.); 3 Academy of Romanian Scientists, 54 Splaiul Independentei, RO-050094 Bucharest, Romania; 4 Department of Orthopedics and Trauma I, Colentina Clinical Hospital, 19-21 Soseaua Stefan cel Mare, RO-020125 Bucharest, Romania; danlaptoiu@yahoo.com; 5 Faculty of Mechanics, University of Craiova, 13 Alexandru Ioan Cuza, RO-200585 Craiova, Romania; lgruionu@gmail.com; 6 Faculty of Medicine, Lucian Blaga University of Sibiu, RO-550169 Sibiu, Romania; horatiu.dura@ulbsibiu.ro

**Keywords:** bone defect, bone regeneration, Mg-based scaffold, angiogenesis, osteogenesis, patient-adapted strategy, manufacturing technology, additive manufacturing, artificial intelligence

## Abstract

Treatment of bone defects resulting after tumor surgeries, accidents, or non-unions is an actual problem linked to morbidity and the necessity of a second surgery and often requires a critical healthcare cost. Although the surgical technique has changed in a modern way, the treatment outcome is still influenced by patient age, localization of the bone defect, associated comorbidities, the surgeon approach, and systemic disorders. Three-dimensional magnesium-based scaffolds are considered an important step because they can have precise bone defect geometry, high porosity grade, anatomical pore shape, and mechanical properties close to the human bone. In addition, magnesium has been proven in in vitro and in vivo studies to influence bone regeneration and new blood vessel formation positively. In this review paper, we describe the magnesium alloy’s effect on bone regenerative processes, starting with a short description of magnesium’s role in the bone healing process, host immune response modulation, and finishing with the primary biological mechanism of magnesium ions in angiogenesis and osteogenesis by presenting a detailed analysis based on a literature review. A strategy that must be followed when a patient-adapted scaffold dedicated to bone tissue engineering is proposed and the main fabrication technologies are combined, in some cases with artificial intelligence for Mg alloy scaffolds, are presented with examples. We emphasized the microstructure, mechanical properties, corrosion behavior, and biocompatibility of each study and made a basis for the researchers who want to start to apply the regenerative potential of magnesium-based scaffolds in clinical practice. Challenges, future directions, and special potential clinical applications such as osteosarcoma and persistent infection treatment are present at the end of our review paper.

## 1. Introduction

Nowadays, most modern treatments are patient-specific because clinicians elaborate on individual therapies and diagnoses. They have to clarify and understand many parameters for each case, and Neural Networks, Machine Learning, or Big Data become important tools in biomedical statistical approaches [1]. A main concern that can be identified in the orthopedic field consists of the standardization of surgical procedures, which was initially a requisite in order to perform certain interventions correctly. However, with time, more personalized approaches were required, specifically in several fields—reconstructions after tumors (orthopedic oncology), reconstructions after advanced deformities (diabetic, neuropathic foot and ankle surgery), reconstructions after the revision of failed endoprosthetic implants (revision surgery), etc. On the other hand, in the case of oncological treatments, individualized strategies such as antibody or adapted chemotherapy led to an increase in patient survival and much more positive results compared to conventional approaches. Patient-adapted implants must also follow this trend in orthopedics. The bone defects generated by tumor surgery [2], spinal surgery [3], and revision arthroplasty [4] have different geometries, and only seldom they can be included in an existent standard [5]. Supplementary, in some cases, collateral damage, such as bone defects, negatively influences implant stability, and patient-adapted pieces must be developed. Luckily, three-dimensional (3D) printing technologies make the manufacturing of personalized implants possible by analyzing and processing the medical images of bone defects [6]. Another important factor that influences the treatment success is the alignment technique, which has to be individualized as a function of implant position and soft tissue tension [7]. If this procedure is applied in joint arthroplasty, a physiological load transfer must be ensured during the joint movement [8]. Many researchers believe that optimizing, adapting, and transforming the standard cases for each patient’s anatomical requirements must be a direction to be followed in the orthopedic field due to its promising results in the possibility of treating different shapes of bone defects and to provide limb salvage in the case of accidents or tumor resection [9].

It is well-known that bone exhibits a high regenerative property, which permits bone strength restoration in the same fashion as it was before the injury [10]. Unfortunately, in the case of critical-sized defects resulting from traumatic accidents, tumor resection, congenital malformation, or infection, the healing process does not spontaneously occur [11]. The classification of bone defects divided them into cavitary and segmental defects. The first ones do not interfere with limb biomechanics but can negatively influence prosthesis implantation and osteogenesis. The last mentioned are much more dangerous because they interfere with the limb biomechanics, affecting the entire bone’s structural stability and viability [12]. The treatment must consider an increased risk of limb amputation and morbidity in these cases. Critical-size bone defect management remains a continuous challenge for patients and surgeons, and interdisciplinary approaches involving the participation of orthopedic, vascular, and plastic surgeons combined with material science engineering activity must be considered. The concomitant use of standard implants and patient-adapted scaffolds can be considered a starting point in segmental bone defect treatments, which often require many surgeries with an unpredictable bone union, a high possibility of infection, and difficult techniques such as Ilizarov or Masquelet that are hardly tolerated by the injured patients [13]. Supplementary, the critical size defects are usually associated with important limb injuries and patient difficulty in social and professional life integration, which shows the immediate necessity to develop new and innovative treatments.

The development of different biomaterials is an important improvement made in the critical-size defect treatment direction. Firstly, metallic materials alloys such as stainless steel, cobalt-chromium (Co–Cr), and titanium (Ti) were extensively used in clinical practice due to their excellent mechanical properties and high biocompatibility [14]. Unfortunately, they are inert materials, and important drawbacks such as metal ions’ and particles’ release due to material wear or corrosion processes can be directly linked to metalosis, allergies, or even poisoning [15]. Another important disadvantage can be named to the fact that Young’s modulus has a higher value in comparison with that of the human bone, and the stress-shielding effect often occurs. In most cases, secondary surgery for implant removal is necessary after the bone tissue healing process is completed [14]. A much more beneficial effect can be foreseen in the case of biodegradable biomaterials that do not require supplementary surgeries. It was put in evidence the fact that the small diameter molecules resulting from material degradation regulate the bone regeneration microenvironment, facilitating new bone formation [16]. Different degradable materials, such as natural or synthetic polymers, were tested and clinically applied over time. To name a few approved by the US Food and Drug Administration (US FDA), one can mention the natural polymer collagen-based grafts (Collagen-graft^TM^ and OssiMend^TM^) that are a combination of calcium phosphates and porous bone mineral with bovine collagen, respectively [17]. Other materials are synthetic complexes such as polycaprolactone (PCL), poly (L-lactic acid) (PLLA), poly (glycolic acid) (PGA), and other compounds [16]. Even though the biodegradable polymers were considered a suitable solution for critical size bone defect management, they have a decreased mechanical strength, and one of their by-products determined the apparition of inflammatory reactions and hindered the new bone formation [18]. One of the best solutions proposed by the researchers consists of magnesium-based (Mg) alloy implants and scaffolds. These materials are characterized by an elasticity modulus value close to the human bone, superior biodegradability, and high biocompatibility [19]. Very few adverse reactions were reported because Mg is an essential element in bone osteogenesis [20] (Table 1) that supplementary facilitates the formation of new blood vessels [21]. In the present days in clinical practice, Mg alloys are met in screw [22], plate [23], and scaffold [24] manufacturing, and their beneficial effects for fracture fixation and bone defect repair are already proven [25]. One of the Mg-based implant producers is MAGNEZIX^®^, certified in 2013 by the European Union for screws used in hand or leg surgeries. The Mg–Ca–Zn alloy commercialized by the Korean producer RESOMET^®^ was approved in 2016 by the Korean Food and Drug Administration (KFDA) for hand fracture treatment. Subsequently, China developed high-purity Mg screws for avascular necrosis of the femoral head [26], and a more recent Mg–Nd–Zn-based alloy (JDBM) was patented by Shanghai Jiao Tong University, proving excellent results after many in vitro and in vivo studies based on scaffolds were performed. The high-speed degradation process of Mg-based implants remains a significant challenge [27]. Much research is dedicated to maintaining the implants’ mechanical properties for the entire bone healing period, reducing hydrogen emission, and diminishing the influence of the highly alkaline environment due to Mg ions’ presence [17,28].

**Figure 1:**
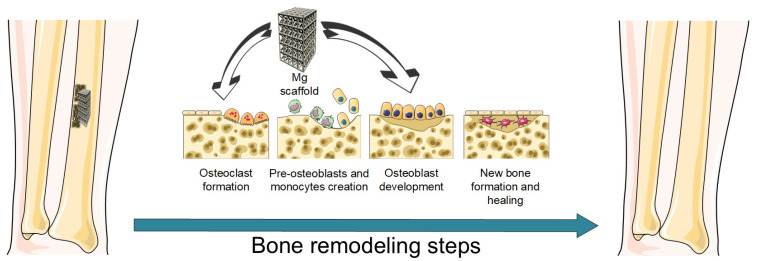
Bone defect treatment based on Mg alloy scaffolds. The figure was generated using images assembled from Servier Medical Art, which are licensed under a Creative Commons Attribution 3.0 unported license (https://smart.servier.com, accessed on 25 August 2023).

Many material and structural parameters must be considered to design an adequate scaffold dedicated to bone tissue engineering (BTE) [29]. Scaffolds can be defined as “artificial temporary platforms” used to repair, support, and populate a specific structure with living cells. In practice, two-dimensional (2D) scaffolds, which exhibit the advantage of better interaction between cells and biomaterials, are used [30]. However, they show limited applicability in only restauration of the plane and small damaged body regions, so the necessity to develop three-dimensional (3D) scaffolds can be foreseen. The last-mentioned structures can mimic natural tissue or organ behavior and sustain their functionality (Figure 1). The main attributes that are required for scaffolds are: a liable structure for cell differentiation, adhesion, and proliferation to sustain the oxygen circulation and nutrients’ dissemination, to ensure biomechanical support adequate for tissue regeneration, and to permit cell and growth factor encapsulation in their geometry to help the damaged part of the body regenerate quickly [31]. Usually, donor or host cells are collected and multiplied in ex vivo conditions and then introduced into the geometrical structure of the scaffolds. Supplementary, it is indicated that the scaffold material has an active character and sustains the neo-tissue formation around it, facilitating its integration inside the human body. The most important characteristics that must be focused on in scaffold manufacturing are exposed surface area, porosity, mechanical properties, material elemental composition directly linked to cell activation, adhesion, proliferation, and biomaterial decomposition kinetics [32].

The main objective of this review article is to summarize and detail the application of patient-adapted Mg-based scaffolds in the bone regeneration tissue engineering domain to address the challenging problem of bone defects. We provide insight into the Mg alloys’ important influence on bone regeneration by presenting the main biological routes usually followed in bone osteogenesis and blood vessel angiogenesis. Furthermore, we present a strategy for the patient-adapted scaffolds starting from the medical images, design of the elementary cell and scaffolds, the need for mechanical finite element analysis and computational fluid dynamics, material properties, constraints, loads, boundary conditions, ending our presentation with adequate technologies, which can permit, in some cases, the artificial intelligence use. In the last part of the review paper, we address the challenges associated with the rapid degradation of Mg-based alloys and corrosion problems and discuss two innovative applications of scaffolds in oncology and infections.

## 2. Mg Alloys Effects on Bone Regeneration

This section will present the bone healing process and the Mg alloys’ role in initiating and sustaining angiogenesis and osteogenesis in the case of bone defects. This information is important for researchers who design biomimetic and biodegradable scaffolds to adapt their geometry to fit human bone properties better. All the studies presented in this section regarding the effect of Mg-based alloys on enhancing the bone healing process provide important insight and put a theoretical basis for an extended application of Mg in orthopedics. Based on the literature, the mechanisms of Mg ions in hard tissue healing are very complicated and include many cell types, regulators, and signaling networks that are necessary for achieving good bone regeneration. Although many positive effects were investigated and summarized here as provisional theory, there is, unfortunately, still free space for elucidating completely the mechanisms of Mg alloys in sustaining bone healing.

### 2.1. Bone Healing Process

In order to design and analyze patient-adapted scaffolds, it is necessary to understand human bone physiology and structure. As a standard definition, bone can be considered an open-cell composite material containing hydroxyapatite (Ca_10_(PO_4_)_6_(OH)_2_), osteogenic cells, extracellular matrix (ECM) proteins, growth factors, and a complex vascular system [33]. The component cells of the bone contain osteoprogenitor cells derived from mesenchymal stem cells (MSCs), osteoblasts, bone-resorptive cells generated from hematopoietic stem cells (HSCs), osteocytes, and osteoclasts [34,35]. The bone progenitor cells (pre-osteoblasts) are derived from MSCs. Under the effect of different growth factors such as bone morphogenetic proteins (BMPs), fibroblast growth factor (FGF), interleukins, transforming growth factor—β (TGF—β), and insulin- and platelet–derived growth factors (IGF and PDGF), the pre-osteoblasts transform into mature osteoblasts.

A general conclusion, which must be considered when biomimetic and patient-adapted scaffolds are designed, consists of the fact that in the case of cortical bone, there is an important mechanical anisotropy with higher values of mechanical properties along the longitudinal axis. For the trabecular bone, a much broader range can be established [33].

Bone healing consists of three main phases: inflammation, repair, and remodeling. After the bone injury occurs, immune system cells, osteoblasts, and injured cells secrete pro-inflammatory mediators that attract many more immune cells, which generate an inflammatory response at the bone injury place [36,37]. MSCs, fibroblasts, and vascular precursor cells facilitate bone healing, and the repair phase begins [37,38]. This phenomenon is accompanied by the formation of new blood vessels [39]. During the second phase, a shift of the microenvironment towards an anti-inflammatory character is observed. The main driving force consists of macrophage polarization into M2 anti-inflammatory phenotype and the release of growth factors and anti-inflammatory mediators that make the osteoblast-related cells dominant during bone repair [40,41]. The final result is callus formation. In the remodeling phase, the callus structure is transformed due to osteoblast and osteoclast action, and a mature lamellar structure occurs [37,42]. In the case of critical size defects, non-union is frequently characterized by the soft callus’s failure to transform itself into a mineralized bone matrix. In these cases, bone tissue engineering involving different patient-adapted scaffolds becomes necessary to restore the normal function of the bone.

### 2.2. Mg Alloys’ Role in Osteogenesis and Angiogenesis

#### 2.2.1. Host Immune Response Modulation

Magnesium is a biomaterial that can regulate the host’s immune system, create new blood vessels, and promote osteogenesis at the injury site. It can interact with different types of cells during the bone regeneration stages to enhance bone healing [43].

When a scaffold is implanted into a host body, the blood protein and the interstitial fluid are absorbed on the material’s surface [44]. This process leads to hemostatic clot formation due to coagulation. The hemostatic clot is linked to migration, proliferation, and adhesion of immune system cells. The fibrinogen (Fg) is considered a key mediator during this stage that induces hemostatic clot formation and platelet activation. Usually, Mg alloys are associated with the process of Fg adhesion [45,46]. Some studies evidenced that the alloying elements used in Mg-based materials play an important role in the Fg absorption process (e.g., the increase of Zn content determined an inhibition in Fg absorption) [45] since the addition of rare earths (REs) generated an increased absorption process [46]. After this stage, neutrophils are recruited near the scaffold surface and an acute inflammatory response appears. Monocytes migrate at the injury place and differentiate into macrophages [47,48] that play an essential role during the inflammatory phase, generating a much more adequate immune system response [36]. A moderate degradation rate of Mg-based alloys is important in the early-stage debridement process [48]. The size and amount of second-phase particles inside the Mg-based alloys are essential for easy digestion of the macrophage cells and for proper immune system activation [49]. The adaptive immune system is then activated through the maturation of dendritic cells and the activation of T lymphocytes. It was found that Mg, as a Ca antagonist, can regulate the immune body response, directly influencing Ca homeostasis and intracellular free Ca^2+^ ions by generating the maturation of dendritic cells and T lymphocyte activation [50,51] (Table 1).

**Figure 2:**
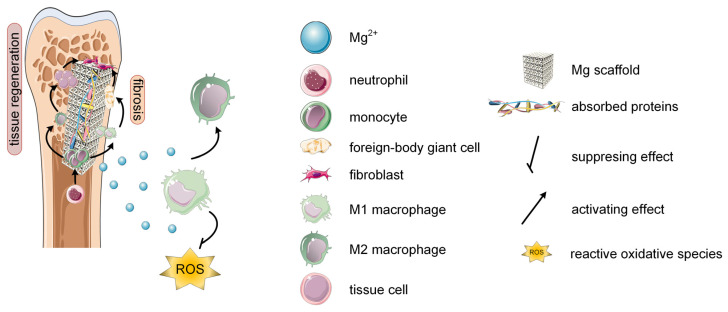
The role of Mg-based scaffolds in promoting the M2 macrophage polarization to generate an anti-inflammatory medium beneficial for the bone-healing process. The figure was generated using images assembled from Servier Medical Art, which are licensed under a Creative Commons Attribution 3.0 unported license (https://smart.servier.com, accessed on 25 August 2023).

As explained in the previous subsection, the transformation of the macrophage cells into the M2 phenotype is very important (Figure 2). Research showed that a low concentration of Mg^2+^ ions and the presence of a low alkaline environment are key factors in the polarization of M2 macrophages [52,53,54]. This fact is beneficial to tissue vascularization [55] and matrix mineralization [56]. The M2 macrophages have a good interaction with MSCs and promote bone regeneration. Due to the Mg effect on macrophage conversion from M1 to M2 phenotypes, the suppression of the inflammatory process is noticed because M2 macrophages generate pro-regenerative factors and anti-inflammatory cytokines that promote the osteogenic differentiation of MSCs [57,58,59] (Table 1). The anti-inflammatory action of the Mg alloys is based on the following mechanisms such as the activation of the P13K–AKT1-signaling pathway, elimination of intracellular reactive oxygen species (ROS), inhibition of NF-kB/mitogen-activated protein kinase (MAPK)-signaling pathway, etc. [54,60,61,62,63,64,65]. As a general conclusion regarding the Mg-based alloy in promoting the M2 macrophage polarization from the M1 state, we can notice that it is of utmost importance to analyze each clinical case and to establish if the treatment route for bone defect amelioration to include Mg scaffolds after tumor resection or infections. The chemical composition, the presence or the absence of coatings, and physical properties such as porosity, topology, and pore size of Mg-alloys have to be carefully analyzed to ensure enough time for M1 macrophage cells to exhibit tissular debridement, microbicidal, and tumoricidal effects. The M2 macrophage polarization should occur only after these steps are complete.

#### 2.2.2. Main Mechanisms of Mg-Based Alloys during the Angiogenesis

In the recent literature, there are only a few studies that underline the Mg alloys’ effect on the revascularization process and angiogenesis [20,54,66,67,68]. After the implantation of the Mg alloys inside the host body, the angiogenesis-related factors such as hypoxia-inducible factor (HIF) [69,70] and vascular endothelial growth factor (VEGF) are upregulated. HIF represents a transcription factor characteristic for angiogenesis and promotes the VEGF transcription under hypoxia conditions [69]. However, VEGF is defined as a growth factor that activates as a stimulus for the endothelial cells (ECs) by binding its receptors and driving the chemotactic and mitogenic responses of ECs. The ECs’ proliferation, adhesion, and migration are sustained, and angiogenesis can be considered successful. Some studies showed that the coatings based on Mg^2+^ ions applied on Ti scaffolds increased the upregulation process of the MagT1 on the surface of HUVECs, conducting to the stimulation of VEGF transcription through HIF-α activation [71]. Another study put in evidence that the Mg degradation products with a concentration between 2 and 8 mM helped in a high amount the expression of VEGFA and VEGFB under hypoxic conditions [72]. Good therapeutic results, such as bone mineralization and regeneration, were reported in [26] to treat femoral head necrosis (Table 1).

**Figure 3:**
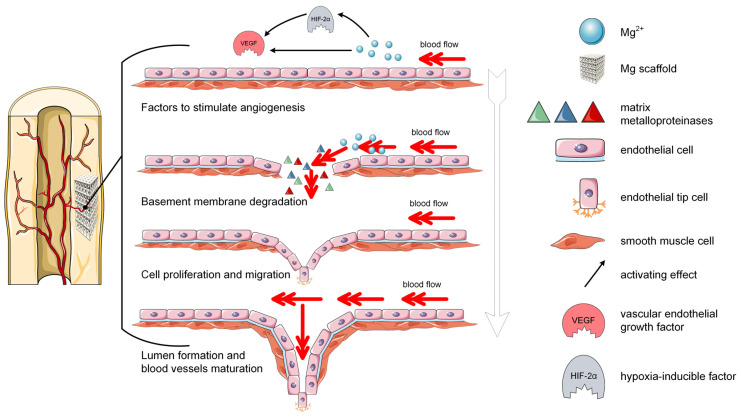
Role of Mg^2+^ ions in angiogenesis and the importance of angiogenesis-related factors (HIF, VEGF). Endothelial cell proliferation and migration, blood vessel formation, and vascular lumen apparition. The figure was generated using images assembled from Servier Medical Art, which are licensed under a Creative Commons Attribution 3.0 unported license (https://smart.servier.com, accessed on 25 August 2023).

The apparition of new blood vessels can be due to the degradation of the basement membrane phenomenon (Figure 3) [71]. The biomolecules of matrix metalloproteinase (MMPs) determine a digestion effect of the basement membrane, and, through EC liberation from the vascular wall, the diffusion and migration are encouraged [73]. In [74], it was shown that 6.25% of the Mg–Zn–Mn extract improved in a high amount the expression of MMP-2. It is well-known that MMPs help the ECs sprouting through VEGF release and bind them to the matrix. After the basement membrane is degraded, ECs are liberated. They proliferate, migrate, and transform themselves into cells, having different functions and geometrical shapes under the effect of angiogenic signals, and new endothelial branches are formed. The literature review demonstrates that Mg promotes the ECs’ proliferation, migration, and tube formation as a function of its concentration [74,75]. It was noticed that the tube formation could be influenced by the effect of Mg^2+^ ions activity by neuropeptide calcitonin gene-related peptide (CGRP), which plays a crucial role in the promotion of EC migration and tube apparition [67,76] (Table 1). Other research [75,77] found that the upregulation of platelet-derived growth factor—BB (PDGF-BB), which is an important indicator of angiogenesis, occurs in the presence of Mg^2+^ ions. After the lumen is completely developed, it will expand under the action of VEGF and Rho-associated protein kinase (ROCK). In this way, the blood perfusion starts making the circulation of nutrients and oxygen possible, together with a downregulation of VEGF expression [77] (Table 1). Development and stabilization of the new blood vessels occur later in the angiogenesis process [68]. It can be noticed that Mg plays an important role in angiogenesis at the injury site, a fact that makes the researchers consider it an adequate material for bone tissue engineering.

#### 2.2.3. Osteogenic Mechanisms of Mg-Based Alloys

The beneficial effect of Mg-based alloys on differentiation, proliferation, and adhesion of osteogenic cells was intensively investigated in the literature [49,52,78,79,80]. They prove to stimulate the alkaline phosphatase (ALP) that is considered an early osteogenic marker, the Runt-related transcription factor 2 (RUNX2) and Osterix (OSX), which are defined as osteogenic regulators, and the late osteogenic markers such as osteopontin (OPN), bone sialoprotein (BSP), BMP2, collagen I (Col-I), and calcium deposition [81,82,83]. One key factor that influences the development of osteogenic-related cells is the concentration of Mg^2+^ ions after alloy degradation. It was established that a concentration between 5 mM and 20 mM is favorable to the development of MSCs, periosteum cells, and osteoblasts [84,85,86]. A higher concentration of Mg^2+^ ions can be considered detrimental by diminishing cell proliferation and inducing cellular death [67,87] (Table 1). Other important features that must be considered when analyzing osteoblast-related cell viability are the medium pH and H_2_ release. At the bone defect site, the environment exhibits an acidic behavior, and transformation into an alkaline medium is beneficial for osteoblast development [88,89,90]. Studies put in evidence that a pH of about 8.5 is beneficial for the osteogenic differentiation of the stem cells [91], while a pH higher than 9 can inhibit cell proliferation and spread [92] (Table 1). Regarding the H_2_ release, it is already known that a rapid degradation process of the Mg-based alloy occurs, and air cavity apparition may impact the cell viability [93,94,95]. Unfortunately, there is a lack of literature regarding the H_2_ concentration effect on osteogenesis, and much more research must be done in this direction. Liu et al. [96] showed that H_2_ emissions significantly inhibited osteoclastogenesis and correlated it with a potential anti-resorption effect of Mg-based alloys.

The adhesion process of the osteoblast is governed by the integrins [97]. Osteoblasts anchor to these binding elements on the cell membrane to ECM. Different literature studies evidenced that Mg-based alloys promote the expression of various integrins, as shown in Table 1 [20,98]. Activating integrin-independent and integrin-dependent intracellular-signaling pathway networks will further govern the osteogenesis process. Research has shown that Mg^2+^ ions mediate the MAPK-signaling pathway, including JNK signaling, ERK1/2 signaling, and p38 signaling, and this fact can be considered of utmost importance during osteoblast differentiation [67,99,100]. Another critical aspect that must be considered when defect restauration is expected is activating the Wnt-signaling pathway [101]. This signal pathway represents one of the essential aspects in bone repair and bone homeostasis, being a wanted target in drug agents such as monoclonal antibodies against Wnt antagonists (i.e., Dkk-1, Midikine, and Sclerostin) [101,102]. Other activations of osteogenesis-related-signaling pathways under the effect of Mg^2+^ ions, such as the Smad-dependent-signaling pathways, were investigated, but their influence still needs further development [103].

A successful bone remodeling must also consider the osteolytic effect of the osteoclasts. Mg^2+^ concentrations, pH of the environment, and H_2_ emission have an important influence on osteoclast formation and viability. It was proved that higher than 25 mM Mg^2+^ ion concentrations determined a decline in osteoclast differentiation since at a concentration of 5 mM, the formation and activation of osteoclasts are sustained [84] (Table 1). Regarding the pH of the environment, it was found that a value between 7.0–7.5 is beneficial for the differentiation and proliferation of the osteoclasts, while a value of 6.8 determines a decrease in the osteoclast bone resorption activity [104]. Supplementary, the H_2_ release proves to inhibit osteoclast formation, as mentioned before [96,105].

**Figure 4:**
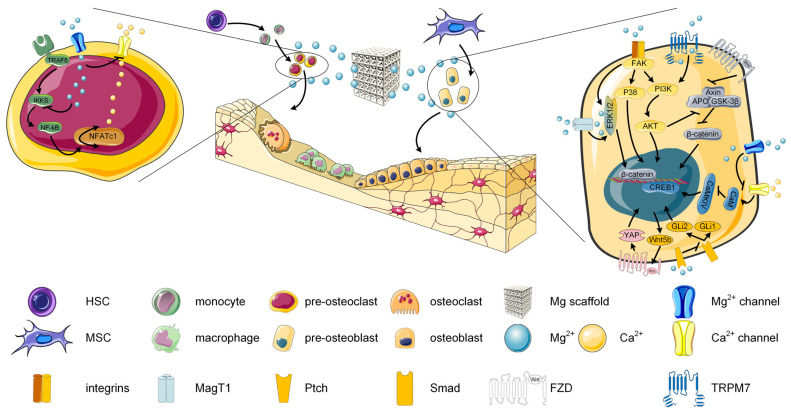
Signaling pathway involved in the osteogenesis process due to Mg effect. Regarding the osteogenic-related cells (right part), the Mg^2+^ ions upregulate the integrin expression and activate the FAK-signaling pathway. The downstream-signaling pathways, including MAPK (p38 and ERK1/2) and P13K/AKT, are activated. Through P13/AKT, the level of GSK3-β phosphorylation is increased, and the Wnt pathway is activated. Mg ions are transported via MagT1, and they promote the ERK1/2-signaling pathway activation and act on TRPM7 with P13/AKT-signaling pathway kinase activity. Intracellular Mg^2+^ promotes osteogenesis through the CaM/CaMKIV/CREB1-signaling pathway. In the left part of the figure is evidenced the molecular mechanism of Mg, which inhibits the function and differentiation of the osteoclasts. Cytokines such as RANKL and M-CSF promote the main functions of the osteoclasts. Magnesium inhibits the NF-kB-signaling pathway, blocks Ca^2+^, and suppresses NFATc1 expression. As a final result, inhibiting the osteoclast marker gene expression due to Mg^2+^ action leads to inhibition of the osteoclastogenesis process. The figure was generated using images assembled from Servier Medical Art, which are licensed under a Creative Commons Attribution 3.0 unported license (https://smart.servier.com, accessed on 25 August 2023).

In Figure 4, the osteogenesis mechanism and the effect of the Mg-based alloys on osteoblasts and osteoclasts is presented.

All the aspects presented in this section prove that Mg-based alloys are a good candidate for patient-adapted scaffold manufacturing due to their beneficial effects on angiogenesis and osteogenesis. Despite the variety of in vitro and in vivo studies existent in the literature, the ideal cellular response after Mg alloy insertion can be just speculated because, as presented in this section, many factors such as rapid speed degradation of Mg-based alloys, environment pH, alloying elements, and concentration of Mg^2+^ ions at the defect site must be considered before the scaffold to be applied from a translational perspective. In Table 1, some literature studies that show experiments regarding Mg alloys’ influence on new bone formation are analyzed.

**Table 1:** Mg alloy’s role in osteogenesis and angiogenesis.

Mg Alloy Effect	Main Role	Event	Alloy/Material	Remarks	Reference
Immune response modulation	Inflammatory activation at the injury site	Protein absorption. Fibrinogen (Fg)—key mediator for platelet activation and clot apparition	Mg-Zn	The Fg absorption was facilitated into the alloy with the lowest Zn content (Mg–1Zn) in comparison with Mg–2Zn and Mg–3Zn alloys	[45]
		Recruitment of neutrophils and macrophage phagocytosis	Mg–Nd–Zn–Zr (JDBM)	A high concentration of magnesium ions determined an M1 macrophage polarization state due to the early degradation of Mg-based alloys that determined the apparition of inflammatory reactions. It was concluded that high concentrations of Mg-based ions determined the early debridement at the injury site	[48]
		Activation of the adaptive immune system of the host	Nanoparticles containing Mg	The nanoparticles, which were enhanced with Mg, generated cell-mediated Th1 and antibody-mediated Th2 immunities after in vitro research. It was confirmed the positive influence of Mg-based materials on the T lymphocyte effect	[51]
	Anti-inflammatory immune microenvironment apparition	Mg action on M2 macrophage polarization	Mg–Si–Ca	The alloy extract showed a significant effect on inhibiting the expression of pro-inflammatory cytokines, proving a reduction of the M1 macrophages	[52]
			Mg–10Gd, Mg–2Ag	The Mg-based alloys determined an increase of the M2 phenotype of macrophages concomitantly with a reduction of the M1 phenotype	[53]
		M2 macrophages and MSCs interaction	Mg–Nd–Zn–Zr (JDBM)	An Mg-alloy scaffold induced a damping effect on the macrophage inflammatory profile simultaneously with chondrocyte differentiation of MSCs	[63]
		M2 macrophages’ role in angiogenesis	Mg–Zn–Ca	The alloy was implanted in a rat femur and showed evidence of early angiogenesis	[54]
	Immunomodulatory signaling pathways	Reduced ROS level	Mg–Nd–Zn–Zr (JDBM)	The Mg^2+^ ions resulted from the Mg–Nd–Zn–Zr degradation determined the apparition of an environment with a reduced number of ROS	[61]
		Activation of P13K/AKT signaling pathway	Mg-based alloy	The Mg^2+^ ions had a beneficial effect on P13K/AKT-signaling pathway activation due to lipopolysaccharide (LPS) inflammatory response decrease	[64]
Mechanisms of Mg alloys during angiogenesis	Apparition of factors that stimulate angiogenesis	Upregulation of hypoxia-inducible factor (HIF) and vascular endothelial growth factor (VEGF)	Mg^2+^ introduced on a Ti scaffold surface	An important upregulation of the MagT1 expression on the HUVECs surface was put in evidence	[71]
			Pure Mg	Mg degradation products with a concentration between 2-to-8 mM promoted the expression of VEGFA and VEGFB under hypoxia conditions	[72]
	Degradation of vascular basement membrane	MMPs that digest the basement membrane and liberate ECs	6.25% Mg–Zn–Mn extract	The expression of MMP-2 was improved, showing evidence for the start of angiogenesis	[74]
	Endothelial cell proliferation, migration, and tube formation	Mg concentration	Mg-based alloy	At a concentration of Mg^2+^ ions equal to 1 mM, the proliferation of HUVECs is improved. This fact remains valid until an Mg^2+^ concentration of 5 mM. At higher than 10 mM concentrations, the angiogenic factor secretions and tube formation are drastically affected	[75]
		Neuropeptides involved in Mg-induced angiogenesis	Mg–Zn–Gd scaffold coated with Ca–P	The scaffold promoted and influenced the CGRP serum in a large bone defect in a canine animal model. The inhibition of CGRP generated a down-regulation of new blood vessel numbers at the defect place	[67]
		Platelet-derived growth factor (PDGF-BB) positive influence on angiogenesis	Mg alloy	A concentration of Mg^2+^ ions between 1 and 5 mM had a good influence on PDGF-BB expression in HUVECs as a function of metallic ion concentration	[75]
		Stability and maturation of the newly formed blood vessels	Mg–Cu	The Mg-based alloy extract determined an upregulation of the endothelial receptor tyrosine kinase TIE-1 and activin receptor-like kinase ACVRL1 in the HUVECs plasma membrane	[68]
Osteogenic mechanisms of Mg-based alloys	State and function of the osteoblast-related cells	Mg^2+^ concentration influence	Mg alloy	An Mg^2+^ concentration between 2.5-to-5 mM was found to increase the osteogenic differentiation of rBMSC cells	[87]
			Mg alloy	A concentration of magnesium ions comprised between 5 and 10 mM had the best effect on ECM mineralization and osteogenic differentiation of hBMSCs. The viability of the cells was drastically decreased when the Mg^2+^ concentration exceeded 20 mM	[67]
		pH influence	Mg–Ga layered double oxide nanosheets deposited on alkali-heat-treated titanium	The osteogenic differentiation of MSCs and autophagic activity were promoted by the alkaline microenvironment with a pH of about 8.5	[91]
			Mg alloy	At a pH between 9 and 10, an important decrease of hFOB 1.19 cells was reached	[93]
		Integrin-dependent cell adhesion	Mg–1Zn, Mg–1Zn–0.5Sn	A promotion effect on the expression of integrin β1 and α1 was achieved	[98]
			Mg–1.0Ca–0.2Si	The alloy extract stimulated the expression of the following integrins: α5, α4, α3, and β1, β5 on the surface of hMSCs	[20]
		MAPK-signaling pathway activation	Mg–Zn	The osteogenic differentiation of BMSCs was sustained by activating the MAPK-signaling pathway, concomitantly with ERK1/2 signaling, JNK signaling, and p38 signaling	[99]
			Mg–1Ca–2.0Sr	In this case, only ERK1/2 was activated, while p38 and JNK pathway proteins were not upregulated	[100]
		Activation of Wnt pathway	Mg-3.5Li-0.5Ca	The activation of Wnt/β-Catenin was achieved, and the osteoblastic differentiation of hBMSCs was attained	[83]
		Activation of Smad-dependent signaling pathway	Mg-1Y/Mg	The Smad-dependent signaling pathway upregulated the expression of BMP2 family members (TGF-β and TGF-β1)	[103]
	Formation and function of the osteoclasts	Mg^2+^concentration influence	MgCl_2_	At a concentration of Mg^2+^ ions of about 5 mM, the formation and activation of the osteoclast were encouraged, while at a concentration higher than 25 mM, the cell viability decreased	[84]
		pH influence	Mg/Mg alloy	The inhibitory effect of material extract regarding the osteoclast activity was reversed after the pH neutralization	[105]
		H_2_ release	-	Different H_2_ concentrations were chosen to study osteoclastogenesis. At 50% and 75% H_2_, the process related to osteoclast-induced BMMCs was inhibited and led to cellular apoptosis	[96]

## 3. Patient-Adapted Strategies for BTE

This section will provide a patient-adapted strategy, which must be followed for personalized implant manufacture and material tests.

The individual regenerative potential of the patient must be considered when a scaffold is designed. It is well-known that many clinical trials are based on adult MSCs to stimulate and help the bone tissue regeneration process. However, unfortunately, the results may vary due to the low reproducibility of the regenerative strategy results and unexpected reactions of the patient tissue [106]. During clinical trials, there are chosen selection criteria for patients that can exclude old persons and patients with associated comorbidities [107] since, for the preclinical analyses, a few healthy and young animals are tested. In this way, the estimation of the final regenerative result of the product could be impaired for old patients and people with different activity ranges.

Usually, the anatomical data of the patient defect is obtained after magnetic resonance (MRI) or computed tomography (CT) investigations are conducted [108]. These procedures generate images of high quality for the hard tissue, and one can distinguish the density variations met at the interface between soft tissue and bone. MRI investigations are suitable for soft-tissue and tumoral expansion analysis. The “gold standard” in patient-adapted scaffolds designed for BTE is represented by CT scans, which are obtained based on the absorption grade measurements of X-rays in the human bone. Based on 2D image results, an intensity map of the bone in the Digital Imagining and Communications in Medicine (DICOM) format is generated by the 3D–CT system. Specialized programs can stack the medical images, and, based on contrast segmentation and the grayscale value of each voxel, a 3D representation of the patient’s bone is achieved. Volumetric representation is involved in obtaining a high-quality 3D reconstruction [109]. Firstly, it uses a 2D segmentation procedure to extract the geometry of the object by selecting a bone region. This operation can be conducted manually on each independent slice or automatically for the entire structure performed [110]. After a set of closed contours is achieved, they are stacked by the program and lead to a 3D solid model. A smoothing operation can be applied to eliminate the residual wiggles from the surface resulting from a previously applied skinning operation. Then, the surface model is obtained, and 3D segmentation is applied to select a tiled surface formed from connected triangles. The model can be transferred for a second analysis in a computer-aided design (CAD) program to generate and optimize a solid model, which can be further processed based on a finite element analysis (FEA) software.

**Figure 5:**
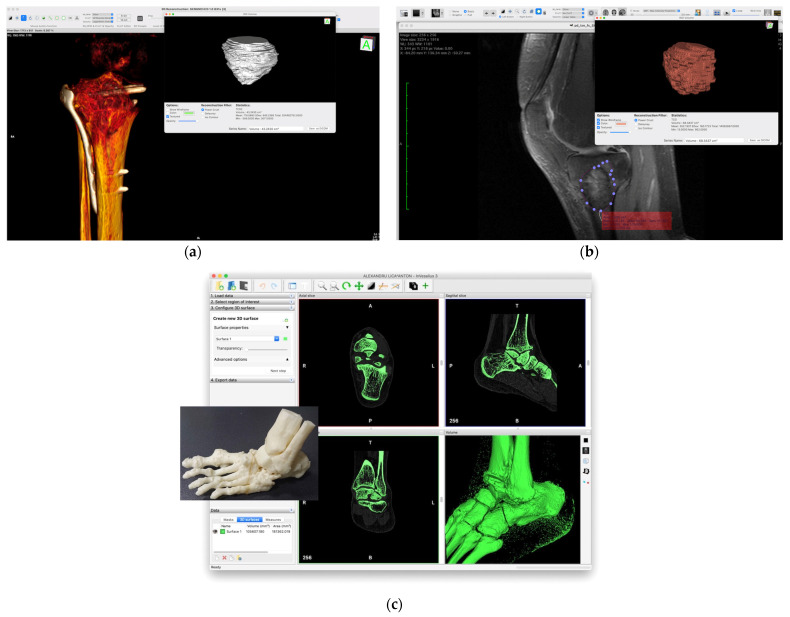
Patient-adapted analysis based on 3D medical image segmentation software using anonymized DICOM images: definition of bone defects volume/topography on (**a**) CT—image; (**b**) MRI image with identification of the bone defect (purple dot circle); (**c**) Patient leg reconstruction (3D-printed model for surgical planning).

To summarize, the following flow is applied: the 3D–CT system saves the 3D image as a DICOM file, then the 3D–CAD system reads the DICOM file and generates a Standard Triangulated Language or Stereolithography (STL) file. Finally, the slicer software converts the STL file into G-code, which is a series of two-dimensional data. In addition, the additive manufacturing system produces 3D objects using the G-code slice data. The reverse engineering strategy presented in this section leads to accurate and stable CAD models, but, unfortunately, the entire process can be time-consuming. This method allows the researchers to isolate the bone defect and obtain a clear delimitation. Figure 5 presents examples that provide insight into how the shape of bone defects can be identified and analyzed from a patient’s DICOM images and the way a part of the patient’s limb can be printed and investigated by the clinicians.

In order to design a patient-adapted scaffold, some structural requirements must be optimized. The first one is represented by its internal structure, which has an important influence on the cell response and mechanical properties [111].

**Figure 6:**
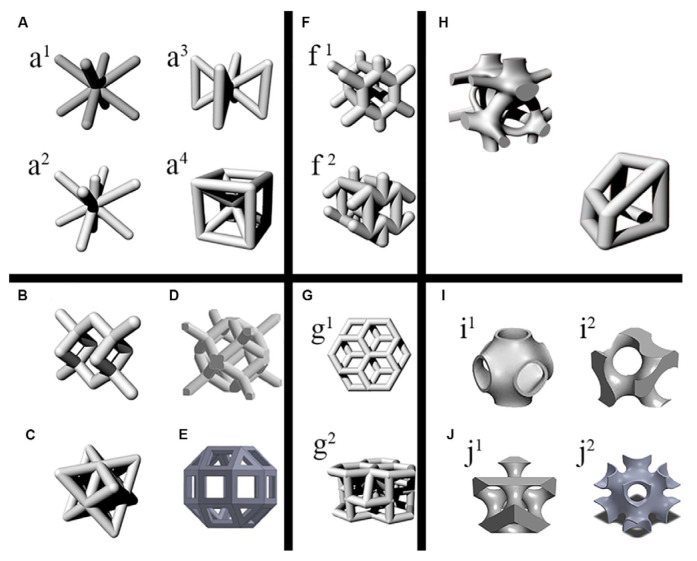
Examples of cell units based on non-parametric and parametric designs: (**A**) Body-centered cubic (BCC) unit and its modified versions (a^1^: BCC unit, a^2^, a^3^: BCC unit with vertical stiffeners, a^4^: BCC unit with pillars); (**B**) Diamond unit; (**C**) Octet unit; (**D**) Rhombic dodecahedron unit; (**E**) Rhombic cube octahedron unit; (**F**) Honeycomb units (f^1^: common structure, f^2^: inverted structure); (**G**) Modified honeycomb units (g^1^: axial view, g^2^: sagittal view); (**H**) Voronoi units; (**I**,**J**) Triply periodic minimal surface (TPMS) units [112] (i^1^: primitive surface, i^2^: I-WP surface, j^1^: diamond surface, j^2^: gyroid surface). The figure is licensed under CC–BY 3.0.

The unit cell represents the basis of the porous scaffold structure from the geometrical point of view. Usually, after the unit cell is designed, the 3D scaffold can be made by replicating it in different spatial directions. Its structure is of utmost importance in establishing adequate mechanical properties of the implants, with values close to those of the human bone and a porosity higher than 50% that will facilitate the fluid flow and the cell seeding and viability. The unit cells of the scaffold can have different shapes [112,113], as presented in Figure 6. Precise control over their characteristics may change the final product’s mechanical properties and porosity and is an aspect that must be considered in BTE.

FEA is a suitable tool that permits the analysis of different factors such as pore diameter and strut size influence on the average mechanical properties. In this way, a specific geometrical structure of the scaffold can be chosen for a particular medical application in which a particular value of stiffness, compressive strength, and elastic modulus are necessary [114].

To analyze the transport property of the nutrients and oxygen and the cell seeding capacity of the scaffold, a computational fluid dynamics (CFD) analysis must be undergone [115]. This investigation is of utmost importance because the fluid velocity through the scaffold geometry provides an idea to estimate the necessary time for cells to adhere at the scaffold surface [116] and to influence the scaffold osteointegration. Supplementary bone remodeling process and hydrolysis could be investigated and upgraded via FEA [117,118,119]. After an optimized structure of the patient-adapted scaffold is obtained based on Boolean operations executed to investigate a perfect match between the defect shape, and the proposed geometry of the scaffold must be performed. If the results are good, the CAD model can be translated to dedicated software linked to a 3D printer as a surface tessellation (STL) file to obtain the final product.

**Figure 7:**
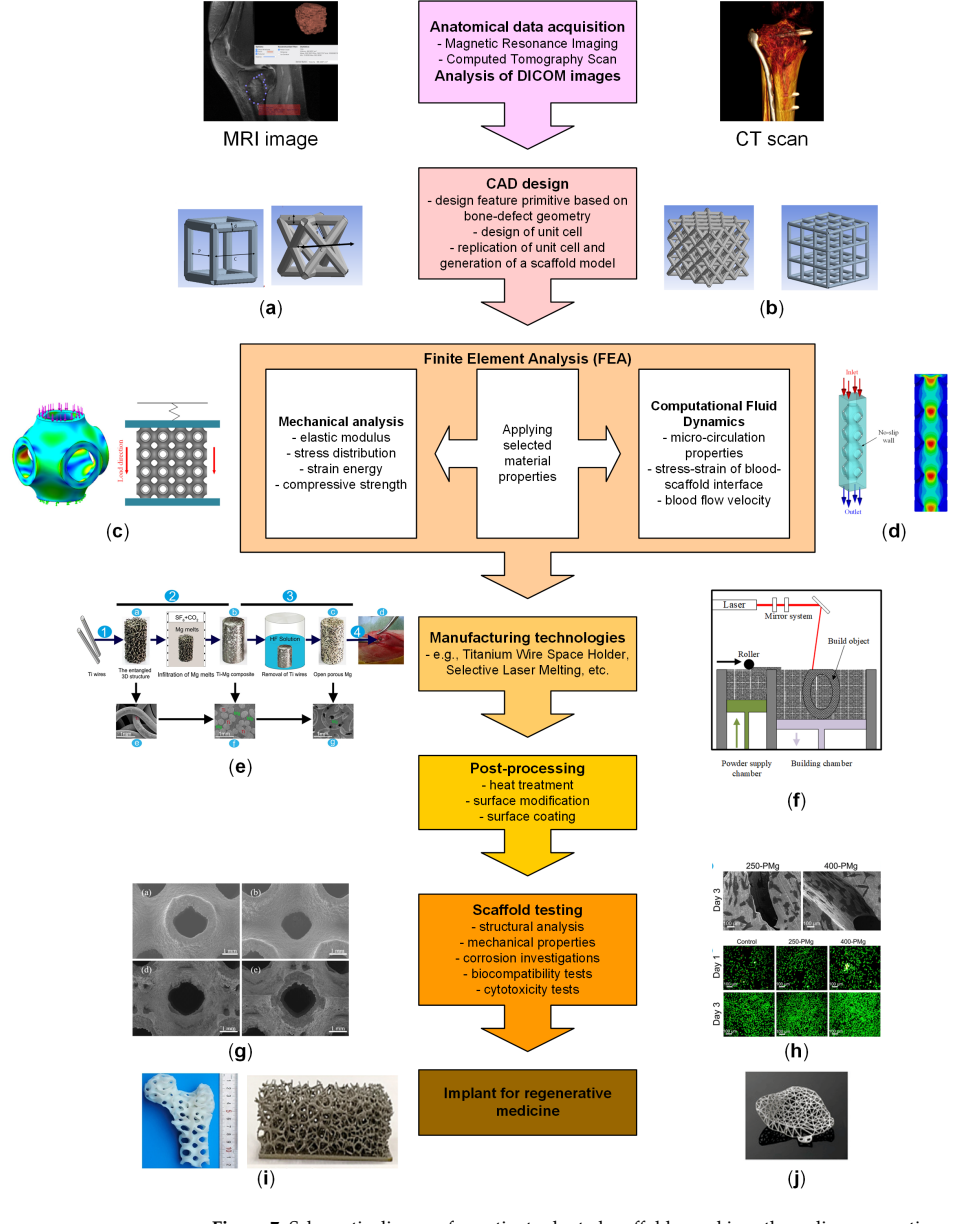
Schematic diagram for patient-adapted scaffolds used in orthopedic regenerative medicine: (**a**) Elementary cells [113]; (**b**) BTE scaffold geometry [113]; (**c**) Mechanical FEA [114]; (**d**) CFD analysis [114]; (**e**) Scaffold produced through titanium wire space holder technology [123]; (**f**) Scaffold produced through selective laser melting [122]; (**g**) SEM micrographs of a porous Mg scaffold [114]; (**h**) Cell morphology after 3 days of incubation represented based on SEM images and viability test performed on MG63 osteoblasts [123]; (**i**) Bone implant and scaffold [120]; (**j**) Porous metallic implant [121]. All figures are licensed under CC–BY 4.0.

Although there are many challenges in the patient-adapted scaffold design, such as bone defect complexity and shape, material properties, biocompatibility level, and the possibility of biomolecule insertion, this technique should be applied soon as an alternative solution to the classical approach to treating critical-size defects [120]. Personalized regenerative medicine will become an important tool to correct bone defects, which rarely can have a standard shape, and to help many patients worldwide [121,122]. In Figure 7, a schematical representation of the processes that are necessary to produce a patient-adapted scaffold is provided. 

## 4. Patient-Adapted Mg-Based Scaffolds Manufacturing Technologies

**Figure 8:**
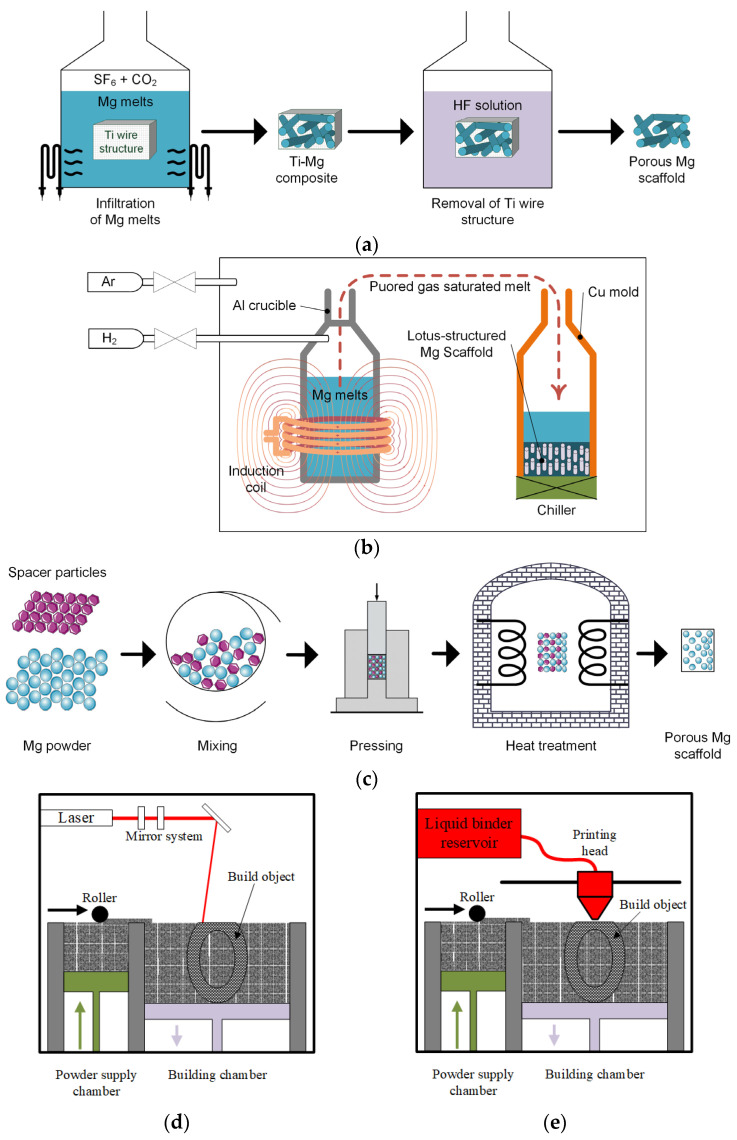
Mg-based scaffold manufacturing technologies: (**a**) Titanium wire space holder method; (**b**) Hydrogen-injection technology; (**c**) Powder metallurgy route; (**d**) Selective laser-melting method [122]; (**e**) Binder jetting technology [122]. Figures (**d**,**e**) are licensed under CC–BY 4.0.

Porous patient-adapted Mg-based scaffolds can be fabricated through different manufacturing techniques (Figure 8). This section will describe some of the main methods used in Mg-based scaffold fabrication with literature examples emphasizing their main material properties, such as mechanical characteristics, biodegradability, and biocompatibility.

### 4.1. Titanium Wire Space Holder Technology

Titanium wire space holder (TWSH) is a novel and innovative technique developed by Jiang et al. [124] and leads to a pipe-like interconnected porous structure. With this method, the pores are the same size, and their diameter is equal to that of the titanium wires. The technique comprises three steps: Firstly, a 3D shape composed of Ti wires is constructed; secondly, this Ti pattern is immersed in liquid Mg, and a titanium/magnesium hybrid is obtained. During the last step, the Ti wires from the hybrid system are dissolved under the action of hydrofluoric acid (HF). In this way, a porous scaffold is obtained. In order to apply the patient-adapted strategy described in Section 3, the bone defect shape must be maintained when the 3D Ti structure is composed, and the bone porosity has to be modeled through different diameter Ti wires.

**Figure 9:**
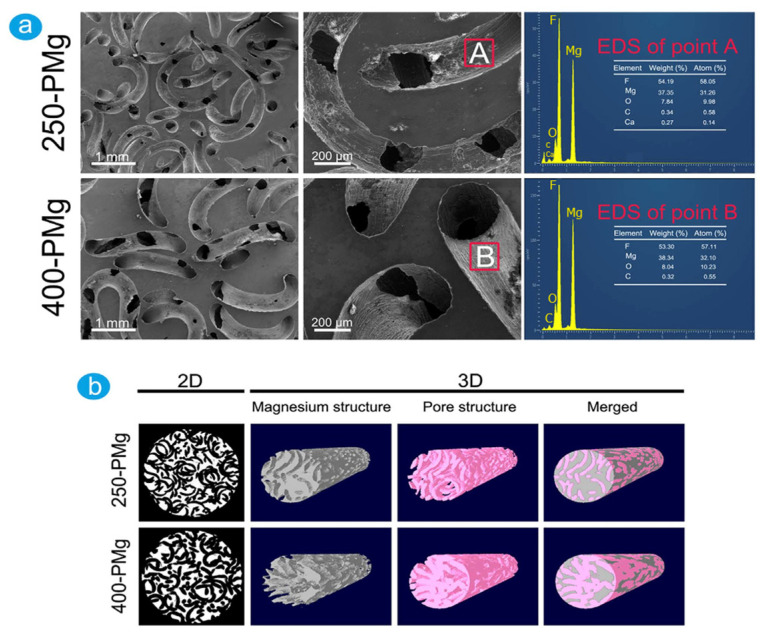
Open porous Mg scaffolds developed through the TWSH method: (**a**) SEM and EDS results; (**b**) 2D and 3D images of scaffolds obtained based on micro-CT scanning [123]. The figure is licensed under CC–BY 4.0.

Jiang and He [124] found that the compressive yielding strength of TWSH-prepared Mg-based scaffolds was between 4.3–6.2 MPa, and Young’s modulus was in the range of 0.5–1.0 GPa since the scaffold porosity was estimated between 43.2% and 54.2%. Cheng et al. [123] (Table 2) developed open-porous pure Mg scaffolds with controllable properties for BTE (Figure 9). The mechanical properties of the scaffolds were regulated to exhibit similar values to those of the cancellous bone through pore orientation changes without any impact on the total porosity. Two porous structures with pore sizes of about 250 μm (250-PMg) and 400 μm (400-PMg), with a total porosity of 55%, were constructed. The Young’s moduli were found to be equal to 2.18 ± 0.06 GPa and 2.37 ± 0.09 GPa, respectively, while the compressive strengths had the values of 41.2 ± 2.14 MPa and 46.3 ± 3.65 MPa, respectively. The authors concluded that these quantities had values close to those of the cancellous human bone (Young’s modulus was between 0.01–2 GPa, and the compressive strength was between 0.2–80 MPa). The in vitro corrosion rates were determined based on immersion tests for 7 days. It was noticed that the 400 μm pore size scaffolds degraded faster than the 250 μm ones. The corrosion rates were estimated at 1.31 ± 0.11 mm/yr and 1.53 ± 0.15 mm/yr, respectively. In order to analyze the cell morphology and attachment, direct tests were combined with indirect cytotoxicity investigations performed on MG 63 cells. It was observed that an increased cell viability was obtained in the case of medium with extracts (*p* < 0.05). In addition, higher ALP and mRNA expressions of ALP, OPN, Runx2, and Col-I indicated an enhanced osteoblastic differentiation induced by the scaffold biomaterial.

The space holder method can be modified as a function of the space holder material. In the literature are reported materials such as carbamide [125], ammonium hydrogen carbonate [126], sodium chloride [127], polymethylmethacrylate [128], saccharose, and sucrose [129], but the use of Ti wires remains the material of choice for Mg-based scaffolds manufacture.

### 4.2. Hydrogen-Injection Technology

Hydrogen-injection technology is considered another method that can be used to obtain porous scaffolds. This method uses melt Mg, which is poured into a crucible in vacuum conditions, while hydrogen with an increased pressure is introduced into the chamber [130]. After that, the melt is heated, and the hydrogen reaches its saturation state. With the help of a water-cooled mold, the melt is unidirectionally upward solidified, resulting in the formation of straight pores due to the supersaturated hydrogen effect.

Gu et al. [131] investigated the degradation, mechanical properties, and cytotoxicity of lotus-type porous Mg-based scaffolds prepared based on the hydrogen-injection method. Scaffolds with an average porosity of 28 ± 1.3% and pore mean diameter of 170 ± 19 μm were obtained. The authors performed uniaxial compression tests at a constant nominal strain rate of 2 × 10^−4^ s^−1^ at 23 °C. A difference between the compressive yield strength of compact Mg (110.3 ± 8.5 MPa) and porous pure Mg (23.9 ± 4.9 MPa) was found before immersion in 150 mL of simulated body fluid (SBF) at 37 °C. In addition, it was observed that the porous Mg samples presented a slower compressive strength decay with the immersion time extension in comparison with the compact ones. Regarding biodegradability, it was observed that after 250 h of immersion, the compact Mg lost about 60% weight since, for the porous sample, this value was about 10% weight loss. It was concluded that a reduced corrosion rate characterizes the porous Mg scaffold. The pH variation curves indicated a rapid increase during the first 72 h, followed by a stabilization process at the pH value of nearly 10 at 250 h for both types of samples. Indirect cell experiments were performed on L-929 murine fibroblast cells. The porous Mg extract exhibited reduced cell viability (*p* < 0.05) compared to pure Mg, but according to ISO 10993-5:1999, this value was estimated in the tolerant level of the cellular applications. It was concluded that the hydrogen injection technology led to porous Mg-based scaffolds that are adequate for orthopedic applications.

Regarding the patient-adapted strategy, this method can be successfully applied in the case of small bone defects, but supplementary machining operations are necessary to obtain the desired shape of the scaffold.

### 4.3. Powder Metallurgy

Powder metallurgy can be used by mixing the space holder agents with Mg powder and applying a two-step heat treatment to burn out the space holder and generate a sintering process of the pressed powder to design porous Mg-based scaffolds successfully.

Yazdimamaghani et al. [132] prepared multilayer-coated pure Mg scaffolds. The coating was comprised of polycaprolactone (PCL) and gelatin (Gel) reinforced with bioactive glass (BaG) particles. The scaffolds were obtained by mixing Mg powder with particle sizes between 150–300 μm with carbonate hydrogen ammonium particles used as space holders. The mixed powders were pressed at 400 MPa. To eliminate the space holder, a heating procedure at 175 °C was applied and followed by a sintering treatment for Mg scaffolds performed at 600 °C. The porosity volume fraction of the scaffolds was found to be about 35–40%. The degradation behavior was analyzed based on SBF immersion tests for 14 days. A weight gain process was put in evidence in the case of coated samples due to precipitate formation, and the weight loss began more slowly than in the case of uncoated scaffolds. A difference in the pH values between 9.6 for pure Mg samples and 7.7 for the complex-coated scaffolds was evidenced after 3 days of immersion. After 7 days of immersion, the uncoated samples were fully degraded since 87% of the coated scaffolds remained intact. It was concluded that the developed coating was suitable for orthopedic scaffold applications in which the implant degradation speed must be strictly controlled.

Seyedraoufi et al. [133] manufactured porous Mg-4 wt.% Zn and Mg-6 wt.% Zn-porous scaffolds based on powder metallurgy. They used the carbamide CO(NH_2_)_2_ with variable volume content of 15%, 25%, and 35% with a medium diameter particle size of about 350 μm as a space holder. A pressing pressure of 100 MPa was followed by a first heating treatment at 250 °C for 4 h to dissolve the space holders, and then by four consecutively applied heat treatments with temperatures between 500 °C and 580 °C for 2 h. It was observed that Young’s modulus and the compressive strength of the porous Mg–Zn scaffolds decreased proportionally with the increase in porosity. In the case of the heat treatment applied at 550 °C, Young’s modulus values decreased from 6000 MPa (Mg-4 wt.% Zn) and 7500 MPa (Mg-6 wt.% Zn) at a 20% porosity to about 2800 MPa (Mg-4 wt.% Zn) and 3500 MPa (Mg-6 wt.% Zn) at a porosity of 45%. In the same conditions, the compressive strength values varied between 60 MPa (both alloys) and about 15 MPa (both alloys). The temperature of 550 °C was established to be the optimal one because the other chosen values lead to low mechanical parameters.

The powder metallurgy route can be easily applied to obtain patient-adapted geometries if personalized molds, in which the powders are pressed, are correctly designed. The space holder material must be completely eliminated for biocompatibility reasons, and the choice of the heat temperature parameters must be carefully performed to achieve the desired result.

### 4.4. Laser-Perforation Technology

Another technology applied to manufacture porous Mg-based scaffold is laser perforation. The scaffolds are constructed based on a laser-perforation system that consists of a programable laser machine that acts on Mg ingots. This method is considered simple, and a structure with homogenous and interconnected pores is obtained.

Geng et al. [134] prepared coated Mg-based scaffolds based on the above-mentioned method. They used a multifunctional laser-processing machine with a pulse frequency of 1–10 Hz and width of 0.3 ms, exhibiting an active output power of 100 W. The porous scaffolds were coated with a β-TCP active layer through a chemical process after an alkali-heat pretreatment was applied. The mechanical properties of the honeycomb-like and round-pore scaffolds were measured based on compression tests. From the stress–strain dependence, it was obtained that at a variable porosity between 42% and 50%, the elasticity modulus and strength had values between 0.4–0.6 GPa and 8–12 MPa, respectively. The authors observed that the scaffold’s mechanical properties had values almost equal to those of the cancellous bone. The biocompatibility of the developed scaffolds was tested through adherence and proliferation of human osteosarcoma cells (UMR106). It was noticed that after 3 h of cell incubation, the cell viability obtained in the case of coated Mg scaffold extract was comparable to that measured on control samples. Fluorescence micrographs showed adhesion of UMR106 cells on β-TCP-coated Mg scaffolds since, in the case of uncoated scaffold, the cells exhibited a reduced viability. The degradation behavior was investigated through pH measurements after the scaffolds were immersed in Hank’s solution. pH values lower than 8 were found during measurements after 1 month of immersion. In the second and third weeks, a slight decrease in pH was observed due to apatite formation and OH^-^ ion consumption. Overall, the pH increases directly proportional to the release of Mg ions during the degradation process of Mg and the pH was about 9 after 2 months of immersion. The entire study proved that laser perforation is a suitable method for scaffolds used in BTE, and bioactive coatings are favorable for the osteogenesis process.

When patient-adapted scaffolds are designed based on this method, it is preferable that the Mg ingots attain the bone defect shape through different additional machining steps.

The technologies, which are presented in Section 4.1, Section 4.2, Section 4.3 and Section 4.4, encounter difficulties in scaffold manufacturing that exhibit complex structures and shapes and may induce defect apparition (Table 2). However, these methods lead to porous structures that are adequate for BTE, but the precise control of the scaffold shape to perfectly fit the patient bone defect is difficult to obtain. In some cases, machining operations are necessary. Another problem that can be identified consists of the fact that the chemical composition and pores’ shapes and sizes may differ from one scaffold region to another and negatively impact the mechanical properties, corrosion behavior, and biocompatibility.

### 4.5. Laser-Based Additive Manufacturing Technologies

In the patient-adapted scaffold manufacturing domain, one of the most-used technologies is additive manufacturing (AM). It has the following advantages: simplification of molding procedure and reduced time for production cycles; manufacturing of complex porous structure with shapes obtained directly from patient DICOM files, which is difficult to achieve through other technologies; energy and material economy, environmentally friendly method, and reduced costs for labor and production steps. During this time, research on AM techniques dedicated to Mg-based products has encountered important difficulties due to Mg’s high reactivity and pyrophoric character, which are directly linked to an unpredictable oxidation process. The main raw materials for Mg-based AM are powders and wires, and in the case of both, the surface energy has an increased value and may lead to unwanted combustion [135].

Based on AM technology, it is very easy to optimize scaffold properties such as permeability, porosity, mechanical stiffness, and strength [136,137]. It can be noticed that random porous structures do not have homogenous mechanical and biological properties at the micro-scale level, as presented in the previous subsection, and strut twisting or bending can occur in some unwanted situations [138]. However, based on AM technologies, interconnected porous structures with superior properties can be manufactured to perfectly fit the patient’s bone defect and generate an optimum cellular response.

One of the most-used 3D printing machines is laser-based and operates with the help of laser stimulation, which is capable of bonding fluid medium or material powders. The most adequate laser technology for Mg-based scaffolds is the selective laser-melting (SLM) method.

**Figure 10:**
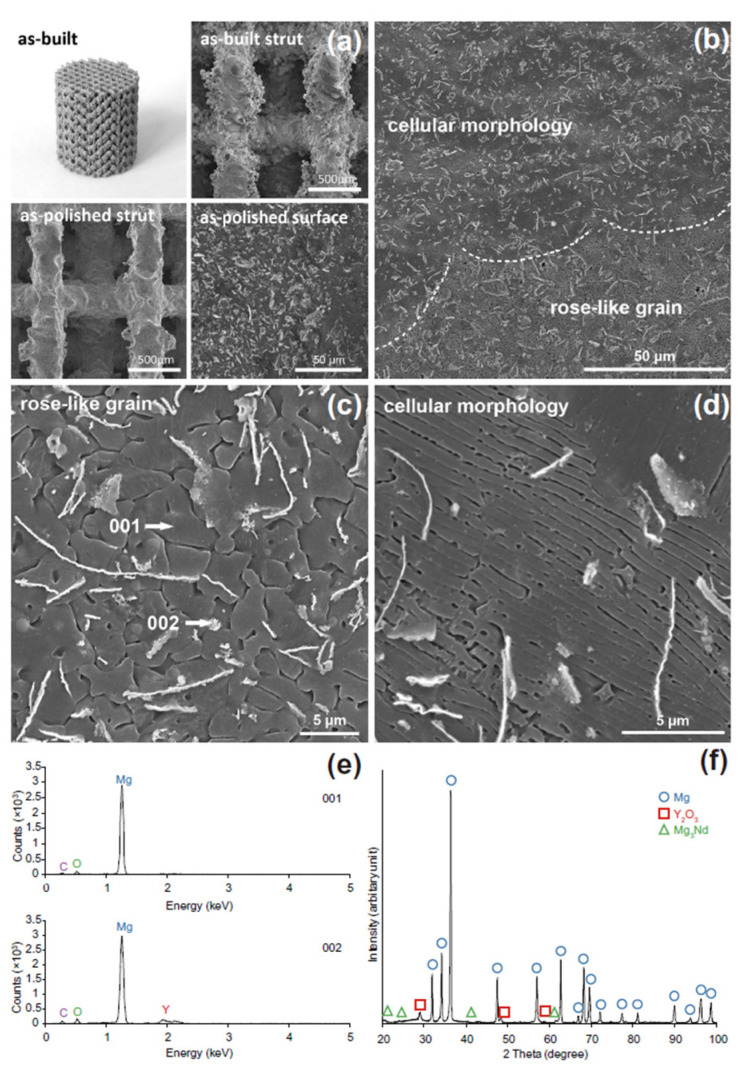
SLM-produced biodegradable porous Mg scaffolds: (**a**) Surface morphology and elemental analysis; (**b**) SEM representation of melt pool; (**c**,**d**) Cellular morphology; (**e**) EDS analysis; (**f**) XRD analysis [139]. Reprinted from [139] Copyright (2023), with permission from Elsevier.

SLM consists of melting and fusing the Mg powder in a predefined pattern within a layer-by-layer configuration. The generated 3D CAD model is divided into layers ranging between 20 μm and 100 μm that are spread at each step with a powder particle bed. A contour outline of the part geometry is formed, and the powder inside this outline is melted using the laser beam. A cooling process is applied to finish the respective layer structure. The process is repeated until the desired 3D geometry is obtained. Li et al. [139] manufactured Mg–Y–Nd–Gd–Zr (WE43) porous scaffolds exhibiting a diamond unit cell based on SLM technology (Figure 10). The porosity of the scaffolds was established through experimental microstructure investigations to be about 64%, with a strut average size of 420 μm and a pore size diameter of 600 μm being in good accordance with the designed values. In order to determine the mechanical properties of the scaffold, compression tests were performed on an Instron machine (10 kN load cell) with a crosshead speed of 2 mm/min. The Young’s modulus was found to be between 0.7–0.8 GPa, being in concordance with properties reported for the trabecular bone (*E* = 0.5–20 GPa). It was concluded that the topology of the porous structure exhibited interconnected pores with a high-porosity grade and precise control of cell geometry. The scaffolds had an adequate in vitro degradation process, characterized by the fact that they maintained their structural integrity after 28 days of immersion. Regarding the pH during the first 3 days, it increased from 7.4 to 8.1 and then gradually decreased. The distant pH kept its values in the biological range of about 7.4, and in the case of longer immersion times, the local pH values were slightly higher than those determined for the distant pH. Supplementary, after 4 weeks of immersion, about 20% volume loss was reported. The scaffold biocompatibility was tested based on MG-63 cell viability. A reduced cytotoxicity of less than 25% was reported. The authors concluded that the WE43 scaffolds manufactured through SLM can be successfully applied as osteointegration implants.

Liu et al. [140] investigated the effect of laser processing variables on the porosity and mechanical properties of an Mg–Ca scaffold produced through SLM. They found that the scaffold porosity and surface morphology were highly dependent on the laser energy input. At an energy density between 875 J/mm^3^ and 1000 J/mm^3^, a porosity between 18.48% and 24.60% was obtained. The microhardness of the samples was determined, and it had values between 60 HV and 68 HV. The authors concluded that the porous Mg–Ca exhibited a higher microhardness than cast pure Mg (35.36 HV) and SML-made pure Mg (52 HV). They demonstrated this affirmation based on the Hall–Petch equation. In addition, a compressive test was applied, and a variation of the ultimate compressive strength (UCS) between 5.18 MPa (laser energy of E = 625 J/mm^3^) and 45.75 MPa (E = 1125 J/mm^3^) was observed in horizontal compression conditions. The sample elastic modulus had values between 0.518 GPa and 0.973 GPa for horizontal compression and at the values mentioned above of laser energy. Regarding the longitudinal compression test, different values were achieved: UCS between 50.91 MPa and 111.19 MPa and Young’s modulus between 0.592 GPa and 0.954 GPa for the same values of E, as mentioned before.

Yang et al. [141] developed a system based on SLM technology to process Mg powders. At laser energies varied between 6 J/mm and 12 J/mm, different values for the samples’ Vickers hardness were obtained as follows: The lowest values of about 42.5 HV for 6 J/mm and 12 J/mm since a maximum value of about 48 HV was determined for 8 J/mm. The sample degradation was studied for probes printed at a laser energy of 10 J/mm through the immersion test in SBF. It was noticed that the pH of the solution increased directly proportional to the immersion time, achieving a value higher than 10 after 60 h immersion. Based on experimental results, it was concluded that the developed system leads to the fabrication of Mg parts with good mechanical hardness and biodegradation behavior. Matena et al. [142] compared selective laser-melted Mg and Ti implants coated with polycaprolactone (PCL). The Mg scaffold had a pore size of 600 μm since the Ti-based scaffolds exhibited a pore size equal to 250 μm. The degradation behavior of Mg scaffolds was analyzed based on the immersion test on Sorensen buffer solution (0.1 M, pH 7.4, 37 °C), and a mass gain was detected in the case of PCL coated sample after 3 days. Then, a predominant decrease of about 100.5% mass development was measured concomitantly to an increase of pH at about 8 after 25 days of immersion. However, for the uncoated samples, a mass development decrease of about 98% and a pH increase higher than 9 were measured. The scaffold’s biocompatibility was investigated based on murine GFP osteoblast cell viability tests. It was found that the Mg scaffolds exhibited an increased cell viability in comparison with the Ti alloy ones.

Supplementary analyses [143,144] are detailed in Table 2. SLM can be considered adequate for the fabrication of scaffolds, which entirely respects the patient’s anatomy. In accordance with the research enumerated in this section, the SLM technology represents the method of choice for many researchers who want to develop implants for BTE. Unfortunately, the process involved in the fabrication procedure is slow and expensive, and the probability of warping or non-uniform heat distribution may interfere with scaffold properties. The manufacturing parameters, such as laser power, must be carefully checked and adapted following the biomedical application because, as presented above, they may impact the scaffold porosity, mechanical behavior, and degradability speed.

### 4.6. Powder-Bed Inkjet Additive Manufacturing Technology

To overcome the disadvantages of the SLM procedure combined with the developed high temperature during the process, the powder-bed inkjet 3D technology is used. The classical method based on the powder’s binding phenomenon through chemical reaction and adhesion mechanisms with a polymeric liquid or solid binder can be modified to be adequate for porous Mg alloy scaffold manufacture.

Salehi et al. [145] developed a binderless jetting procedure for Mg–Zn–Zr alloys based on capillary-mediated properties. This method consisted of a single-phase solvent made especially for Mg–Zn–Zr alloys with the main function consisting of MgO dissolution formed on the outermost layer of Mg particles with no binder components, and any solute constituent was involved at the 70% solvent saturation level during the part building step. The built samples were left for 1 h to dry, and then they underwent different sintering processes made at temperatures between 535 °C and 610 °C in isothermal conditions for 5 h followed by furnace cooling at room temperature. The mechanical properties of the samples variated as follows: The ultimate compressive strength (UCS) had values between 4.86 ± 0.39 MPa (535 °C) and 84.26 ± 4.70 MPa (610 °C), and the elastic modulus started from 2.117 ± 0.026 GPa (535 °C) and reached 13.392 ± 0.019 GPa (610 °C). It was concluded that the sintering temperature, pore size, and densification procedure had a major impact on the mechanical properties’ variations. Another analysis that was performed consisted of a holding time increase from 5 h to 60 h at an optimal sintering temperature of 573 °C, which led to an interconnected open-porous structure (apparent porosity of 29% and pore size of 15 μm) with Young’s modulus of 18 GPa and compressive strength of 174 MPa. The authors concluded that the developed AM procedure is adequate for scaffolds dedicated to BTE because the mechanical properties obtained at the optimal sintering temperature were comparable to those of the cancellous bone. It can be noticed that this capillary-mediated assembly process of powder particles eliminated the necessity of polymeric binders by maintaining the initial composition of the powder and involving a single-phase solvent. In this way, and by controlling the sintering temperature, patient-adapted scaffolds can be precisely manufactured by respecting the mechanical characteristics of trabecular bone.

### 4.7. Indirect Additive Manufacturing Technology

Indirect additive manufacturing techniques based on infiltration can be used in the 3D patient-adapted scaffold fabrication. As in the case of other presented AM methods, a 3D CAD model is produced. After that, a positive template made from different materials is printed and infiltrated with NaCl paste. Heating and sintering treatments are applied to remove the template’s material and increase the density of the NaCl negative pattern. Liquid Mg is cast into the obtained pattern, and, finally, the NaCl is dissolved, and a Mg-based scaffold with the same architecture as that designed in the initial CAD file results [122,146,147].

Nguyen et al. [137] developed topologically ordered porous Mg scaffolds based on the NaCl template. They analyzed the dimensional accuracy, porosity, and surface roughness based on scanning electron microscopy, micro-computed tomography, and confocal laser-scanning microscopy. The designed scaffolds had strut sizes of 0.6 mm, 0.8 mm, 1.0 mm, and 1.2 mm, as well as pore sizes of 1.4 mm, 1.2 mm, 1.0 mm, and 1.2 mm. To evaluate if the scaffolds were manufactured in accordance with the initial CAD structures, the strut size was analyzed. A maximum increase of 8.3% in strut size was noticed for 0.6 mm structures, since for 1.2 mm strut size samples, a deviation of 2.5% was obtained. Regarding the NaCl templates, major changes were observed in the case of 0.6 mm strut size scaffolds, and the lowest ones were evidenced for the 1.2 mm strut size samples. When liquid Mg was infiltrated, an increase in the strut size was considered. A maximum increase of 8.3% occurred for the smallest strut size since a minimum value of 2.5% was noticed for the largest strut size (1.2 mm), as in the other measurements. The surface roughness was about 10.17 ± 1.8 μm for all the investigated samples. Porosity measurements led to a value of 73.9 ± 1.6% for 0.6 mm strut size and 32.8 ± 0.6% for 1.2 mm strut size. It was concluded that this indirect additive manufacturing technology was linked to an increased level of accuracy in scaffold production and conducted to highly order porous Mg-based scaffolds based on low-cost materials and equipment.

Lin et al. [148] investigated the mechanical behavior and corrosion properties of hybrid scaffolds. Initially, they prepared Co–Cr scaffolds with a pore size of 700 μm and total porosity of 80% and then infiltrated these structures in an Mg–Al–Zn (AZ31) liquid alloy at 750 °C and inert atmosphere. The mechanical properties were determined based on compression tests performed before and after the degradation tests. The Co–Cr/AZ31 composite scaffold average stiffness shifted from 18.3 GPa to 5.5 GPa, directly proportional to the mass loss, while the yield strength value dropped from 155 MPa (100% of initial AZ31 content) to 54 MPa (0% AZ31 content). Immersion tests made in SBF display an increased degradation rate compared to classical Mg alloys (lower than 10 mm/yr) due to galvanic corrosion apparition. The authors concluded that despite the hybrid scaffold’s good mechanical properties, much research is necessary in order to reduce the galvanic corrosion process and obtain implants with adequate corrosion rates for biological applications.

**Figure 11:**
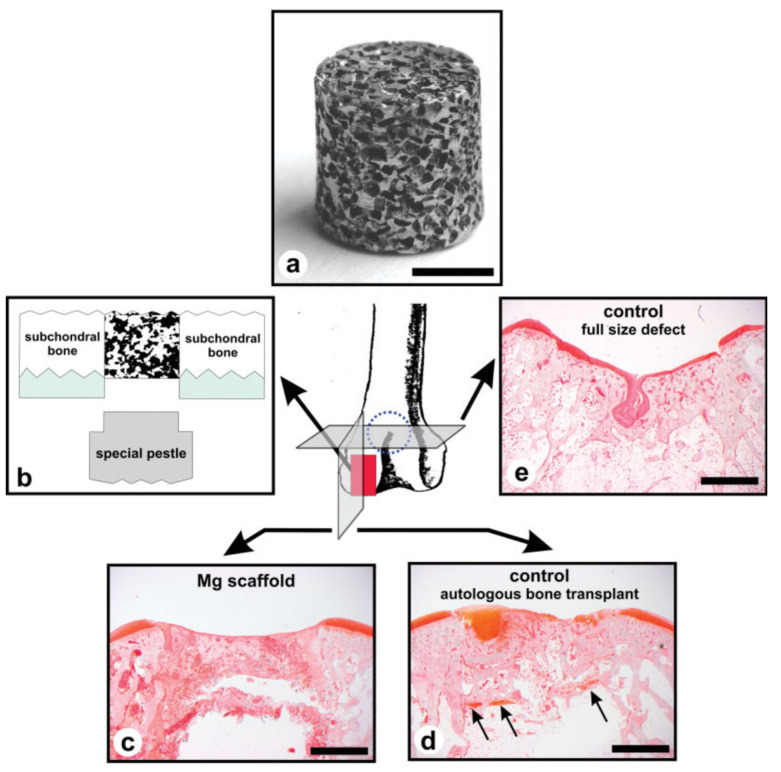
Experimental design of an in vivo study on open-porous Mg–Y–RE–Zr (AZ91D) scaffolds made based on a negative salt pattern molding method; (**a**) Scaffold geometry; (**b**) Implantation procedure; (**c**) Safranin–O-stained longitudinal sections from Mg scaffold site; (**d**) Safranin–O-stained longitudinal sections from autologous bone implant site (black arrows indicate the remnants of the original cartilaginous surface of the reverse implant); (**e**) Safranin–O-stained transversal section of the patellar defect (Scale bar: (**a**) 2000 μm, (**c**–**e**) 500 μm) [149]. Reprinted from [149] Copyright (2023), with permission from John Wiley and Sons.

Witte et al. [149] conducted an in vivo study on open-porous Mg–Al–Zn (AZ91D) scaffolds made based on a negative salt pattern molding method. The authors poured moistened NaCl particles into a core box, generating the NaCl negative template. After that, the pattern was immersed in melted Mg alloy, and the salt particles were eliminated with an NaOH solution. The scaffolds had an average porosity of 74% and variable pore sizes between 10 μm and 1000 μm. For the in vivo experiments, the authors have chosen six New Zealand White (NZW) rabbits. The Mg alloy scaffold was introduced in the left knee of the animals, while in the right knee, an autologous bone cylinder collected from the left patellar groove was inserted to compare the inflammatory reactions (Figure 11). The surgically generated patellar defect was kept empty to emphasize the natural healing effect. After 3 months, it was noticed that a full integration of the autologous bone transplant occurred. Regarding the magnesium scaffold, an appropriate inflammatory response was achieved. Histopathological tests revealed that neutrophile granulocytes are sporadically present at the defect site. The CD3 immunostaining detecting T-lymphocytes evidenced a sporadic presence of these cells with an accumulation localized at the interface between the fibrous capsule and implant. This result was also sustained by the macrophage-specific antibody MAC387 test. The main conclusion of the research was that the scaffolds are suitable for BTE, but supplementary research must be conducted to obtain adequate material coating to reduce the speed of corrosion of Mg-based scaffolds. The biological response obtained in this paper showed the great potential of Mg scaffolds in the bone regenerative process, as presented in Section 2.

The indirect additive manufacturing technique exhibits the advantages of eliminating the Mg powder pyrophoric character, but the geometrical features such as strut size and pore dimensions can hardly be controlled and maintained in the range of those predicted by the CAD models. This method can be successfully used in the patient-adapted scaffold domain, but the manufacturing process has to be carefully monitored.

The methods presented in this section are summarized in Table 2.

**Table 2:** Manufacturing technologies for patient-adapted Mg-based scaffolds.

Method	Advantages	Disadvantages	Mg-Based Alloy Scaffolds	Mechanical Properties	Corrosion Behavior	In Vitro and In Vivo Biocompatibility Tests	Ref.
Titanium wire space holder (TWSH)	Controllable pore-size and scaffold structure; pipe-like structure	The necessity of HF acid use	High-purity Mg	Young’s moduli: 2.18 ± 0.06 GPa (250-PMg) and 2.37 ± 0.09 GPa (400-PMg); compression strength: 41.2 ± 2.14 MPa (250-PMg) and 46.3 ± 3.65 MPa (400-PMg)	Corrosion rates (CR) of 1.31 ± 0.11 mm/yr (250-PMg) and 1.53 ± 0.15 mm/yr (400-PMg)	High cell viability and decreased cytotoxicity for MG63 cells. In vivo tests made on rabbits showed moderate levels of severe inflammatory reactions	[123]
			Pure Mg	Compressive yielding strength of 4.3–6.2 MPa; Young’s modulus in the range of 0.5–1.0 GPa	-	-	[124]
Hydrogen injection	Straight and upward unidirectional oriented pores	The increased cost of casting equipment	Pure Mg	Compressive yield strength of 23.9 ± 4.9 MPa for porous Mg sample before immersion	A weight loss of about 10% occurs after 250 h of immersion in SBF	Indirect cell experiments made on L-929 cells indicated that the developed scaffolds are safe for cellular applications with RGR Grade of 1	[131]
Powder metallurgy	Easy to perform technique; interconnected pore network	Lack of corrosion resistance in the absence of coatings; low mechanical integrity	Complex polymeric coating from PCL and Gel reinforced with BaG particles for pure Mg	-	Difference in the pH values: 9.6 (pure Mg) and 7.7 (coated Mg) after 3 days of immersion. The uncoated samples were fully degraded	-	[132]
			Mg-4 wt.% Zn, Mg-6 wt.% Zn	For the 550 °C heat treatment, Young’s modulus values decreased from 6000 MPa (Mg-4 wt.% Zn) and 7500 MPa (Mg-6 wt.% Zn) at a 20% porosity to about 2800 MPa (Mg-4 wt.% Zn) and 3500 MPa (Mg-6 wt.% Zn) at a porosity of 45%. The values of compressive strength: between 60 MPa (both alloys) and 15 MPa (both alloys)	-	-	[133]
Laser perforation	The structure, shape, and diameter size of the pores are easy to control and modify	Expensive programmable laser processing machine	β-TCP coating on pure Mg	Young’s modulus had values 0.4–0.6 GPa; mechanical strength had values between 8–12 MPa when scaffold porosity variated between 42% and 50%	pH values lower than 8 after 1 month of immersion; then it increased to 9 after 2 months of immersion	After 3 h of UMR106 cell incubation, the cell viability obtained in the case of coated Mg scaffold extract was comparable to control samples	[134]
Selective laser melting data	The powder can be recycled; a rapid solidification of the part can be achieved due to fast cooling and heating cycles; high-density parts with good mechanical properties and adequate biodegradation behavior are obtained	The evaporation process can be detrimental to the Mg powder bed due to its decreased weight, dust explosion danger, high energy-consuming process	Mg–Y–Nd–Gd–Zr (WE43)	The Young’s modulus was found to be between 0.7–0.8 GPa for scaffold samples	pH during the first 3 days increased from 7.4 to 8.1 and then gradually decreased; after 4 weeks of immersion, about 20% volume loss was reported	Reduced cytotoxicity of the scaffold on MG63 cells of less than 25% was determined	[139]
			Mg–Y–Nd–Gd–Zr surface modification based on Plasma Electrolytic Oxidation (PEO) and/or heat treatment	The maximum compressive stress was determined as a function of pore size. It was noticed that PEO-modified structures with big pores had a reduced stress resistance of about 5 MPa in comparison with the ones with small pores (20 MPa)	The big pore PEO surface-modified samples had a lower value of hydrogen emission of 20 mL at 20 days compared to the small pore sample (60 mL at 20 days)	-	[143]
			Mg–Zn–Zr (ZK60)	The hardness of SLM ZK60 porous material was about 0.78 GPa higher than that of cast ZK60 (0.55 GPa)	SLM-produced ZK60 exhibited a higher corrosion resistance in Hanks’ solution, being characterized by a decrease of 30% regarding hydrogen evolution rate and of about 50% in the corrosion current density compared with cast ZK60	-	[144]
Binderless jetting	Faster and easy process	Post-processing steps are necessary due to the limited mechanical performance of the parts	Mg–Zn–Zr	At the optimal sintering temperature of 573 °C, the scaffold Young’s modulus was 18 GPa, and the compressive strength was 174 MPa	-	-	[145]
Indirect additive manufacturing procedure	Elimination explosion danger due to the volatile nature of the Mg	The geometry of pore size and strut can be designed at the macro-scale level; topological mismatch between the as-designed and as-produced Mg scaffolds	Co–Cr/Mg–Al–Zn (AZ31)	The hybrid scaffold mechanical stiffness shifted from 18.3 GPa to 5.5 GPa, directly proportional to the mass loss produced by implant immersion in SBF. The yield strength value dropped from 155 MPa (100% of initial AZ31 content) to 54 MPa (0% AZ31 content)	An increased degradation rate occurred due to galvanic corrosion apparition	-	[148]
			Mg–Al–Zn (Die-cast) (AZ91D)	-	-	In vivo tests made on NZW rabbits. The result was sustained by the macrophage-specific antibody MAC387 test. Low inflammatory reactions for Mg-based implants were reported	[149]

## 5. Artificial Intelligence Techniques for Patient-Adapted Mg-Based Scaffold Manufacture

Artificial Intelligence (AI) is the process of creating computer systems that can simulate human intelligence. These intelligent processes include visual perception, problem-solving, reasoning, language understanding, and learning. AI technologies encompass machine learning, deep learning, computer vision, natural language processing, and more. They are utilized in a diverse range of applications, such as virtual assistants, healthcare diagnostics, and self-driving cars.

Deep neural networks, often simply referred to as deep learning, are a class of artificial neural networks (ANNs) that are characterized by their deep architecture, meaning they have multiple layers of interconnected neurons or nodes. These networks are inspired by the structure and function of the human brain and are particularly suited for tasks involving complex pattern recognition and hierarchical feature extraction. Mitra Asadi–Eydivand et al.’s [150] study utilized an aggregated artificial neural network (AANN) to investigate how three factors: layer thickness, time delay between layer deposition, and how print orientation affected the compressive strength and porosity of porous scaffolds. The goal was to find the best 3D-printing parameters for creating small porous structures, addressing a real problem in BTE. Initially, the particle swarm optimization algorithm was employed to determine the most effective network topology for the AANN. Following this, Pareto front optimization was used to identify the ideal parameter settings to produce scaffolds with the desired compressive strength and porosity. The findings proved the potential of developed strategies in efficiently managing and optimizing the porous-scaffold 3D-printing process, especially for intricate engineering challenges.

A 3D Convolutional Neural Network (3D CNN) is an extension of the traditional CNN, which is designed to process three-dimensional data. It is typically used for analyzing videos, medical imaging volumes (such as CT scans), or other spatiotemporal data. In the field of tissue engineering, mechanical metamaterials are used as scaffolds to replicate the complex mechanical properties of biological tissues. However, conventional design and simulation methods may not be enough to address the challenges posed by geometric complexity, manufacturing issues, or large aspect ratios that create numerical discrepancies. To overcome these challenges, researchers are turning to artificial intelligence (AI) and machine-learning (ML) techniques. In a study, Bermejillo Barrera et al. [151] validated the use of 3D CNNs trained on digital tomography derived from CAD models. These AI-driven models can predict the mechanical properties of novel scaffolds and offer a powerful tool for tissue engineering. The AI-aided or ML-aided design approach represents an innovative advancement in the field of tissue engineering scaffolds and mechanical metamaterials. It has the potential to significantly impact regenerative medicine and offers broader applications beyond tissue engineering.

Support Vector Regression (SVR) is a machine-learning technique used for regression tasks. Unlike traditional regression algorithms, SVR utilizes a different approach. Tourlomousis et al. [152] explored the potential of altering the biophysical properties of biomaterial substrates to control cell shape and, consequently, cell phenotypes. They focused on a previously unexplored dimensional scale in three-dimensional (3D) substrates with precisely tunable porous microarchitectures at the cell’s operating length scales (10–100 μm). The study demonstrated the creation of high-fidelity fibrous substrates using a specialized manufacturing technique called melt electrowriting (MEW). The collected multidimensional dataset was used to train an SVR algorithm to classify cell shape phenotypes. This technology platform represents a significant advancement in integrated additive manufacturing and metrology for various applications, including fundamental mechanobiology research and 3D bioprinting of tissue constructs tailored at the single-cell level. Other AI techniques are based on cellular automata (CA), which are discrete computational models that consist of a grid of cells, each of which can exist in one of a finite number of states. These models evolve over discrete time steps, where the state of each cell depends on the states of neighboring cells according to a set of predefined rules. Cellular automata are often used to simulate and study complex dynamic systems and phenomena.

The scaffolds manufactured through AM technologies can have an improved quality if intelligent systems, which continuously monitor the melt pool or defect apparition, are controlled by machine-learning (ML) algorithms. Today, based on the internet-of-things (IoT) combined with different sensor integration, automatization procedures, robots’ use, and data analytics, new cyber-physical systems (CPS) are used to digitalize the production stages. Some enterprises have already adopted the so-called cloud technologies, which permit receiving or delivering operations via an intelligent system [153], making the premises of cloud manufacturing (CM) possible. In the framework of Industry 4.0 (I4.0), conventional production advances to the incorporation of decision-making capabilities that are based on dedicated software for systems with multiple sensors in a smart factory medium [154], in which robots perform delicate manufacturing steps without human intervention and assistance [155]. Nowadays, Industry 4.0 is considered revolutionary [156] as it is based on smart systems that have the capabilities of self-monitoring and self-structuring [157]. These intelligent systems can substitute human command so the production chain can be autonomously controlled, exhibiting a high grade of automation [158]. A cloud-based facility is implemented to choose the proper online diagnostic adapted for each application [159], and the sensors and tools of the systems are commanded through the cloud. This type of smart-monitoring process is suitable to reduce the risks associated with workpiece and tool damage by linking the device to a CPS based on sensors and IoT, and in this way, the productivity of the process increases [160]. The digital twin technology (DT) is defined as the opportunity, which consists of intelligence and digitization inclusion in the classical material-processing steps [161].

Regarding AM technology, a CPS can be developed and incorporated to provide a prompt response to market necessities in real-time situations and enable the digital value chain [162]. It is well-known that data-driven procedures have made important progress in AM. However, process parameter optimization is still considered a key challenge for future industrialization of AM methods [163,164]. A main problem identified in the literature is the inherent manufacturing defects of 3D printed complex parts that occur during the AM process and sometimes can be characterized by an inferior quality compared with parts made through other technologies [165]. The disadvantage of many post-processing steps is covered by hybrid manufacturing (HM) techniques that are defined as a combination of subtractive (SM) and additive manufacturing technologies [166]. Through HM, the part quality produced based on AM can be increased by eliminating the residual stresses during the 3D-printing procedure [167]. To accomplish the standard requested geometrical tolerance for AM parts, SM steps are necessary. In addition, in eliminating the support materials, an HM workstation can be involved, and the amended production steps are quick and easy to perform [168]. Artificial intelligence (AI) is suitable to solve the challenges associated with 3D printing and to relate the software integration in an adequate manner to address real-time scenarios [169]. Additive manufacturing technologies are prone to automation, but supplementary connectivity, collection, and data-sensing improvements can be foreseen [170].

**Figure 12:**
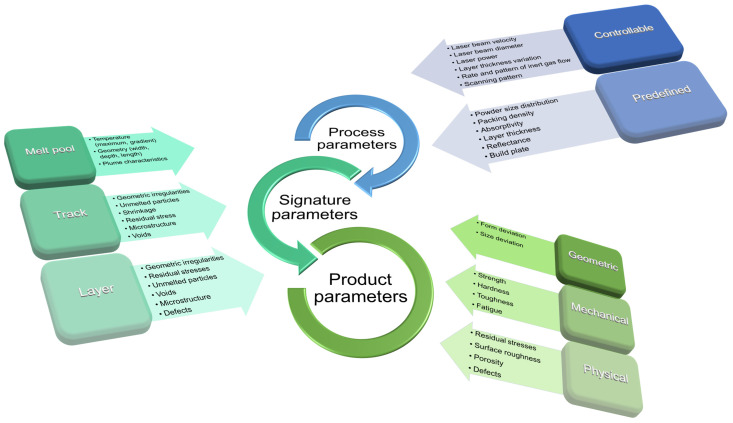
Hierarchical organization of process parameters, process signature, and product qualities in accordance with Mani et al. [186] theory.

The main problems associated with AM parts are the geometrical deviations due to surface defects such as increased roughness, distortion, warping, balling, and sub-surface defects such as material porosity [171]. In some cases, delamination or cracking can occur in the case of coated parts, and residual stresses can be induced during the AM technology in different parts’ areas. The above-mentioned defects are considered an important drawback for AM implementation in large-scale production. The melt pool geometry is responsible for the quality and shape of the deposited track, directly related to the part microstructure, defect morphology and size, and mechanical performances [172]. It was established that a combination of various parameters, such as chamber characteristics [173], build environment conditions [174], laser deposition parameters [175], machine specifications, and material morphology [176], could have an important influence on the melt pool geometry. So, as a direct consequence by understanding and controlling the role of each parameter, an improvement of build structure can be achieved. One of the most-used technologies for Mg-based scaffolds is powder bed fusion (PBF). In order to monitor this process, suitable sensing devices should be identified and used. McCann et al. [177] analyzed the main types of defects that occur during PBF and proposed a proper sensing methodology. In the case of porosity and balling, they found the ultrasonic testing method adequate [178] since, for overheating or cracking, acoustic emission spectroscopy was involved [179,180]. The authors noticed that surface roughness or dimensional accuracy can be estimated based on X-ray tomography [181,182], while fusion defects, PBF irregularities, melt pool fluctuation, and pore formation can be easily seen based on optical coherence tomography [183]. Other studies [184,185] proposed infrared imaging and pyrometry for overheating analysis. Mani et al. [186] analyzed the melt pool’s transient thermal behavior because all the defects described above can occur under its influence. The authors underlined the metrology approaches, noticed the correlations between different process parameters and product quality, and developed a proper strategy for in situ monitoring and control activities. They divided the process parameters into controllable and predefined, the process signature into melt pool characteristics, track, and layer, and finally observed the direct relationship with product qualities such as geometric shape, mechanical, and physical properties (Figure 12). Their study is the first one in which the process parameters and their signatures are logically linked to product quality in a general manner.

Vlasea et al. [187] established the basis of intelligent control in AM by providing the relationship map between measures and, experimental methods, as well as control strategies for PBF technology. They proposed an organizational structure used for control strategies by defining the measurand as the parameter information based on adequate measurement technologies and transformed into the data stream, which is used for post-process analysis, feedback process control, and in situ process monitoring. The control strategy contained four parts as follows: pre-processing for predictive control, in situ defect identification, in situ continuous feedback control, and signature control. The pre-processing step exhibits a multi-layered architecture, which takes into consideration a digital pre-processing stage dedicated to 3D topology design and the input parameter optimization for obtaining the desired configurations. Regarding the in situ defects or fault detection, the controller finds discrete events and, based on corrective action, avoids some process instabilities. The most common meet process errors are due to the non-uniformity of the powder layer [188], powder exhaustion [189], powder contamination, and interactions between super-elevations and re-coater. Other sources for process errors can be considered related to unintended porosity of the green product, increased surface roughness, balling defects [190], material ejection in the melting process, and high shrinkage and curling conducted under the effect of the residual stresses. The defects were addressed as follows: in the case of powder spread quality, an imaging system such as a charge-coupled device (CCD) camera combined with dark-field illumination is used since, for pore or surface roughness, thermal investigation is involved [191]. The in situ continuous feedback control is based on laser cladding and laser surface alloying industries. In SLM technology, feedback control was used to analyze the melt pool parameters [185] and plasma experimental measurements [187]. All the analyses were performed based on thermal measurements made with the help of CCD, CMOS, or infrared (IR) cameras and proportional-integral-derivative (PID) controllers. The signature-derived control is performed by involving simulations or plant models, which are based on in situ feedback signals for non-measurable physical quantities’ estimation. The simulations are always made in parallel with the controller or the look-up algorithms become dynamically accessible to the user. Finally, part quantification should be conducted through an adequate control scheme based on the material structure, while geometry analysis, surface finish, or porosity analysis should be conducted. Vlasea et al. [187] considered this a research opportunity, which can be considered of utmost importance in the industrial field.

Machine learning can be involved during the five stages of the AM process, such as design, performance and process optimization, in situ monitoring and controlling, post-process operation monitoring and controlling, and testing and validation steps [192]. The main challenges met in practice are the lack of common data format and standards, which sometimes make ML integration difficult. The control must not be exclusively based on visual information acquisition because mistakes due to sparkling or increased brightness can be made. As a direct consequence, dedicated sensors that function and provide non-visual information must be involved. In this way, high in situ assistance is offered, and the AM procedure can be controlled in a correct manner. Chen et al. [193] summarized some methods dedicated to the SLM process. They found that the main characteristics, which must be followed in the process are the molten-pool signal, sound and temperature signals, powder layer, slice thickness, deformation of produced parts, and scraper system vibration. As mentioned in this section, non-contact pyrometers, high-speed or near-infrared thermal CMOS cameras, photodiodes, optical coherence, or acoustic emission can be successfully used to monitor the manufacturing process. The result consists of a big data flow, which needs a high memory space for storage and powerful workstations and servers for analysis. The defect detection can be made through deep learning, and a real-time processing procedure based on high-sampling frequency combined with high-pixel analysis is considered vital. From the literature, one cannot find a feasibility confirmation of the monitoring method or a standard procedure for corrective actions or feedback control. Regarding the SLM procedure, the trial-and-error method remains the most-used research technology. The authors concluded that the development of in situ monitoring possibilities can successfully promote the large-scale application of SLM technology. Based on AI and ML, it is much easier to establish a closed-loop feedback system, which uses a large number of process parameters and involves a high number of physical phenomena such as thermal dynamics, fluidic dynamics, and rapid solidification of melted material. The post-processing methods for AM can include neural networks (NNW) or genetic algorithms (GA). The NNW technology was used in the process model generation step, while the second one controlled the polishing parameters. Finally, the prediction of surface roughness was made based on the ML regression method [194]. By combining the AI with AM, the post-processing step of AM parts can be made in an automatic manner. In this way, the input data related to the part-manufacturing method can be incorporated, and expected output responses for the post-processed components can be obtained.

It will be of great interest to include machine intelligence in the AM process to provide autonomous path planification, defect detection, in situ monitoring, sensing, and controlling of the melt pool, as well as to establish a link between these parameters and the printing procedure. Much more automatization of the process can be achieved through machine-to-machine (M2M) communications, and the application of DT, AI, or ML to optimize the entire process. 

## 6. Challenges, Potential Clinical Applications, and Future Trends of Patient-Adapted Mg-Based Scaffolds

The most important challenge in a direct relationship with patient-adapted Mg scaffolds is linked to the increased speed of the corrosion rate [195]. Precise control is vital to hinder the scaffold’s premature failure [196]. Different alloying elements, surface modifications, biocompatible coatings, and grain refinement can be successfully applied to address this drawback [197,198,199]. The crucial Mg alloy interface challenges are related to the adverse effects of hydrogen evolution during the degradation process and decreased mechanical properties. It is well-known that a physiological limit for the hydrogen evolution rate was set to be unharmful at a concentration lower than 0.01 mL/cm^2^/day [31]. Another concern related to Mg-based scaffold manufacturing is linked to corrosion product-induced inflammation, and much research must be conducted to discover highly biocompatible alloys or coatings [200]. Antoniac et al. [201] described the present solutions for controlling the corrosion rate, hydrogen gas emission, and Mg ion concentration in detail. We will shortly summarize the main methods. One of the most-used techniques to decrease the magnesium corrosion speed is the microstructural modification of the surface, such as pulsed electron beam treatment that improves the surface mechanical properties and induces grain refinement concomitantly with surface impurity removal and, as a drawback, has the apparition of crater defects. Another surface modification is laser surface melting, which determines the apatite apparition, enhances mechanical properties, and reduces the porosity grade. Unfortunately, this method is usually related to stress-crack apparition. Surface mechanical attrition and shot peening were used to increase the mechanical properties and wear resistance, but a decreased degradation rate and surface contamination were reported. The surface modification class includes laser-shock peening, which permits increased control over the surface morphology with high wear resistance and good mechanical properties, but no important improvements regarding the decrease of the alloy corrosion rate were reported. Other methods used to reduce and control the Mg-based scaffold corrosion rate are based on physical deposition coatings such as sputtering that is linked to high-quality coatings and improved corrosion resistance, thermal evaporation, and ion plating, which is characterized by a high deposition rate and improved corrosion resistance. In addition, pulsed laser deposition with a low temperature of the substrate and high deposition rate that is associated with high costs and low-quality deposed films is used. Dip coating suitable for the large and complex shape of the coatings, spin coating (which leads to a uniform coating with very good degradation behavior), chemical vapor deposition that is adequate for complex geometry but have a delamination risk, and spray coating associated with increased antibacterial resistance and low degradation rate are also utilized. The chemical conversion coating class includes other technologies presented in the literature that are used to control the Mg alloy corrosion speed. To name the most important methods used to reduce and control the Mg-based scaffold corrosion rate, the following can be considered: the acid and alkali treatment with low cost and high degradation rate exhibiting poor quality coatings and anodization that is related to increased corrosion resistance, high biocompatibility, and apatite formation, but expensive and characterized by a non-uniform deposition process accompanied by crack apparition. Another chemical conversion coating is obtained through electrodeposition with a high corrosion resistance and variable pore size, but sometimes the coating is fragile. Dense and uniform coating is obtained through electrophoretic deposition, but poor adhesion can occur. The micro-arc oxidation procedure is linked to high-quality coatings, promotes apatite apparition and increased corrosion resistance, and has excellent mechanical properties and high antibacterial behavior. Another method that must be mentioned is the hydrothermal treatment, which leads to thin film coatings with decreased corrosion rate, but it is considered inadequate for long immersion times.

An important problem identified in the literature is the complexity grade and intricate shape necessary for the scaffold to treat different bone defects. It is well-known that the degradation of Mg-based scaffolds strongly depends on the implantation site and surface-to-volume ratio of the implant. Establishing a direct connection between these variables is difficult, but the scaffold degradation process should be guided to offer the bone the best mechanical and biological conditions to heal and regenerate.

In the orthopedic field, the scaffolds can be subjected to stress corrosion cracking (SCC) apparition, being continuously inside the patient’s body under combined actions of stress and corrosive medium. To control the SCC phenomenon and diminish its effect, incorporating elements such as Mn and REs, control of texture, and grain size can be applied. This aspect should be considered as a future trend by respecting the biocompatibility range for each metal. In some cases, mechanical properties must be enhanced to provide the bone enough time to heal and maintain the scaffolds’ integrity for a longer time at the defect site [202]. We can also identify the need to develop adequate manufacturing techniques for Mg-based scaffolds with variable porosity, controlled mechanical properties, and microstructure. Supplementary, long-term clinical studies to analyze the biodegradation rate, mechanical stability, and biocompatibility of Mg-based scaffolds dedicated to bone defects should be performed to increase clinicians’ interest in temporary implants in comparison with permanent ones. The lack of standardization for test protocols must be addressed because the key aspects mentioned above must be followed according to the particular guidelines when a 3D patient-adapted scaffold is designed.

A potential clinical application of patient-adapted Mg-based scaffolds could be considered in bone oncological diseases such as osteosarcoma treatment protocol. The anti-cancerogenic effect of magnesium was put in evidence in many research papers. Qiao et al. [203] performed in vitro and in vivo studies and proved the inhibitory effect of Mg against ovarian cancer cells. Another study by Yang et al. [204] observed that rabbit hepatocellular carcinoma cells and murine breast cancer cells were destroyed by Mg-based implants associated with magnetic hyperthermia therapy. In addition, Zan et al. [205] found that Mg wires with good mechanical properties and a reduced corrosion rate of about 1 mm/year inhibit the cellular growth rate of MG63 and U2–OS cells in a proportion of about 99%. The mechanisms analyzed directly with the reduction of cell viability were the medium pH, hydrogen emission, and the effect of Mg^2+^ ions. Many publications considered an antitumoral effect of highly alkaline media, but unfortunately, the experiments are hardly reproducible when in vivo conditions are considered. The authors noticed that a pH value between 7.4 and 8 did not influence the apoptosis of U2–OS cells. Usually, cancer cells are associated with an increase in the content of ROS. A study [206] found that H_2_ emissions are linked to the elimination of hydroxyl radicals. It was observed that OH^-^ had the most increased cytotoxic effect in cancer cells, and through its elimination, the high content of ROS produced by U2–OS and MG63 cells can be controlled. Another factor analyzed is the role of Mg^2+^ ions against cancer cells. We have already presented in Section 2 the beneficial effect of magnesium ions on osteoblast differentiation and osteoclast inhibition at a concentration between 5 and 20 mmol L^−1^. At higher concentrations between 10–20 mmol L^−1^ of Mg^2+^, an increased apoptosis rate of MG63 and U2–OS cells associated with a reduced proliferation process was observed. We can conclude that an important potential clinical application can be related to the treatment of bone cancer-suffering patients by concomitantly offering them the possibility to regenerate the bone defect and killing the residual cancer cells present at the defect site.

Another potential clinical application for Mg-based porous scaffolds is a combined action of osteogenesis and angiogenesis with antibacterial properties against gram-positive and gram-negative bacteria. In this way, infections can be prevented at the bone-defect site [207]. Saheban et al. [208] prepared porous scaffolds made of Mg–Zn–Ca/Zeolite (Zeo)/Ag based on 1 wt.% Ca, 6 wt.% Zn, 0 ÷ 7 wt.% Zeo, 0.5 wt.% and 1 wt.% Ag using powder metallurgy route and space holder techniques. The authors concluded that the main mechanisms involved in the scaffold antibacterial effect are the release of free radicals, cell function interruption, and cell membrane manipulation [209]. In addition, supplementary mechanisms related to silver, such as binding to DNA and electron transportation, must be considered [210]. Another important investigation that was conducted in the study was the biocompatibility of the scaffold. An important condition for clinical translation is that the scaffold material hinders biofilm formation and bacterial attachment [211].

In future research, we proposed the increase of in vivo studies to determine the correlation between the viability of different cell lines to underline the angiogenic and osteogenic effect of the scaffolds, as well as to determine different alloys’ elemental composition or use of innovative coatings that can enhance the bioactive character of the scaffold.

## 7. Conclusions

In order to produce a patient-adapted scaffold, we need to follow different steps: recording and processing the medical images of the patient, isolation of the bone defect and establishment of the geometrical shape, CAD design of the scaffold, including the unit cell design and its replication to generate the scaffold, mechanical finite element analysis, computational fluid dynamics simulation, manufacturing process, postprocessing steps, and scaffold structural, mechanical, and physical characterization. The manufacturing technology used for patient-adapted scaffolds dedicated to BTE must be chosen in good accordance with the bone defect size and localization. Technologies presented in Section 4.1, Section 4.2, Section 4.3, Section 4.4 and Section 4.7 can be chosen if the pore size and strut dimensions are not of utmost importance in bone regeneration at the injury site. In cases where a homogenous chemical structure and geometrical uniformity of the pores are necessary, classical 3D printing methods such as SLM (Section 4.5) or binder jetting (Section 4.6) can be used. We can conclude that the chosen technology must be completely in accordance with the biomedical applications and the patient’s necessities.

In patient-adapted scaffold production, the implication of AI during the manufacturing steps can lead to better mechanical properties and net and almost perfect geometrical shape or size for the final product to fit perfectly into the patient’s anatomy.

Considering the important role of Mg-based alloys in bone healing, the 3D scaffolds used for bone defect treatment will have great potential in the orthopedic field. They regulate the macrophage polarization process, promote new bone formation with an increased accent on proliferation, differentiation, adhesion, and extracellular matrix secretion of osteogenic-related cells, and play a vital role in the angiogenesis process by controlling endothelial cell behavior. Supplementary, as described in Section 2, Mg ions can exhibit unique properties in avoiding abnormal osteoclast apparition and bone resorption at the defect site. In the literature, there is a lack of in vitro studies that analyze the signaling pathways and phenomena that occur at the cell levels, and much research must be conducted to understand the mechanism of Mg ion actions inside the highly corrosive medium that exists in the animal model bodies. Some authors presented the beneficial effect of Mg alloys in cancer cell death and their important antibacterial action against different pathogen strains, while others suggested that adequate alloy composition must be adapted as a function of the biomedical application, bone defect shape, and size to reach optimal results. Due to the high potential of Mg-based alloys, many medical devices will be certified and launched on the market in the future. In addition, the 3D-designed Mg scaffolds for orthopedics are a promising subject for future research if the mechanical and corrosion behaviors are well-tuned according to medical applications.

## Data Availability

Not applicable.
